# Characterisation of putative class 1A DHODH-like proteins from Mucorales and dematiaceous mould species

**DOI:** 10.1371/journal.pone.0289441

**Published:** 2023-08-02

**Authors:** Corinne Pinder, Ressa Lebedinec, Tim P. Levine, Mike Birch, Jason D. Oliver

**Affiliations:** 1 F2G Ltd., Manchester, United Kingdom; 2 UCL Institute of Ophthalmology, London, United Kingdom; Universidad Autonoma de Chihuahua, MEXICO

## Abstract

Olorofim is a new antifungal in clinical development which has a novel mechanism of action against dihydroorotate dehydrogenase (DHODH). DHODH form a ubiquitous family of enzymes in the *de novo* pyrimidine biosynthetic pathway and are split into class 1A, class 1B and class 2. Olorofim specifically targets the fungal class 2 DHODH present in a range of pathogenic moulds. The nature and number of DHODH present in many fungal species have not been addressed for large clades of this kingdom. Mucorales species do not respond to olorofim; previous work suggests they have only class 1A DHODH and so lack the class 2 target that olorofim inhibits. The dematiaceous moulds have mixed susceptibility to olorofim, yet previous analyses imply that they have class 2 DHODH. As this is at odds with their intermediate susceptibility to olorofim, we hypothesised that these pathogens may maintain a second class of DHODH, facilitating pyrimidine biosynthesis in the presence of olorofim. The aim of this study was to investigate the DHODH repertoire of clinically relevant species of Mucorales and dematiaceous moulds to further characterise these pathogens and understand variations in olorofim susceptibility. Using bioinformatic analysis, *S*. *cerevisiae* complementation and biochemical assays of recombinant protein, we provide the first evidence that two representative members of the Mucorales have only class 1A DHODH, substantiating a lack of olorofim susceptibility. In contrast, bioinformatic analyses initially suggested that seven dematiaceous species appeared to harbour both class 1A-like and class 2-like DHODH genes. However, further experimental investigation of the putative class 1A-like genes through yeast complementation and biochemical assays characterised them as dihydrouracil oxidases rather than DHODHs. These data demonstrate variation in dematiaceous mould olorofim susceptibility is not due to a secondary DHODH and builds on the growing picture of fungal dihydrouracil oxidases as an example of horizontal gene transfer.

## Introduction

Invasive fungal infections are a global public health issue: 1.5 million deaths are attributed to fungal disease while over one billion people are affected by it [1]. Market-ready novel antifungal drugs are lacking while the vulnerable population size increases and resistance to current therapies rises due to various geo-ecological and socio-economic factors [2, 3]. Olorofim is a new oral systemic antifungal from the novel class of orotomides [4], currently in phase III clinical development for the treatment of invasive aspergillosis (ClinicalTrials.gov Identifier: NCT05101187) [5, 6]. Due to its novel mechanism of action, olorofim has potent activity against azole-resistant *Aspergilli* [7] and organisms intrinsically resistant to other antifungals, such as *Aspergillus* section *Terrei* [8]. Additionally, it has broad anti-mould activity [6, 9], covering some species that currently have limited or no treatment options, including *Lomentospora*, *Scedosporium*, and *Scopulariopsis* spp. and members of the *Rasamsonia agrillacea* species complex [10–13]. The spectrum of activity also extends to dimorphic fungi of the genera *Coccidioides*, *Blastomyces*, *Histoplasma*, *Talaromyces* and *Sporothrix* but not *Candida*. [14–16]. Olorofim also lacks activity against *Cryptococcus* spp. and the Mucorales [17]. Various species of pigmented moulds, termed the dematiaceous moulds, have been subjected to *in vitro* susceptibility testing with mixed results. *Madurella mycetomatis* [18] and *Phaeoacremonium parasiticum* [17] are susceptible to olorofim but *Alternaria alternata* [9] and the black yeast *Exophiala dermatitidis* [19] are not. Most of the aforementioned species are recognised as priority fungal pathogens by the World Health Organisation [20].

Olorofim acts through the selective inhibition of fungal dihydroorotate dehydrogenase (DHODH) [4]. DHODH are ubiquitous flavoenzymes that catalyse the fourth step in the *de novo* pyrimidine biosynthetic pathway. Using a flavin mononucleotide (FMN) cofactor and an electron acceptor, they oxidise dihydroorotate (DHO) to orotate in the production of pyrimidines. DHODH vary in structure, electron acceptor and subcellular localisation: these characteristics dictate their categorisation into one of four classes, summarised in **Table 1**.

**Table 1:** Summary of DHODH.

Class	Form	Electron Acceptor	Localisation	Catalytic base	Species distribution
1A	Homodimer	FMN	Cytosol	Cysteine	Gram-positive bacteria, such as *Streptococcus mutans* [21]; Parasites such as *Leishmania major* [22] and *Trypanosoma* spp. [23, 24]; *Saccharomyces cerevisiae* [25]; Mucorales, predicted [4]
1B	Heterotetramer	NAD^+^	Cytosol	Cysteine	Gram-positive bacteria such as *Bacillus subtilis* [26], *Clostridium oroticum* [27] and *Bifidobacterium bifidum* [28]
1S	Heterodimer	Coenzyme Q, O_2_	Cytosol	Serine	*Sulfolobus solfataricus* [29]
2	Monomer	Quinones	Cell membrane [30] or inner mitochondrial membrane [31]	Serine	Gram-negative bacteria [32]; *Plasmodium* spp. [33]; Plants [34]; Insects [31]; Mammals [35]; Most fungi, including *Aspergillus* spp. [36], *Candida* spp. [37]; Dematiaceous moulds, predicted [4]

Notably, the bacteria *Lactococcus lactis* [38, 39] and *Enterococcus faecalis* [40, 41] harbour both class 1A and class 1B DHODH while the yeasts *Lachancea kluyveri* [42, 43] and *Kluyveromyces marxianus* [44] possess both functional class 1A and class 2 DHODH, though *K*. *marxianus* has not been experimentally verified. Class 2 DHODH interact with the respiratory chain in some but not all species, such as *Anaeromyces robustus* [44].

DHODH is a druggable target for therapeutic development in various species. Teriflunomide inhibits human DHODH [45] for rheumatoid arthritis [46] and multiple sclerosis treatment [47]. A novel human DHODH inhibitor MEDS433, based on brequinar, appears to prevent influenza virus replication [48]. *Plasmodium* DHODH has been validated as a new target for antimalarial development; DSM265 is the first to reach clinical development [49]. Olorofim selectively targets the fungal class 2 DHODH and is predicted to bind in the quinone channel, preventing ubiquinone-mediated reoxidation of FMN and thus inhibiting the oxidation of DHO to orotate [4]. Disruption of *de novo* pyrimidine biosynthesis has pleiotropic effects, altering cell wall composition, increasing vacuolar size, perturbing cell division and ultimately cell lysis [50, 51]. Variation in class 2 DHODH protein sequence has been shown to account for the differences in susceptibility; *Candida* spp. vary at residues important for olorofim interaction compared to *Aspergillus fumigatus* and increased evolutionary distance from susceptible species is concomitant with loss of olorofim susceptibility [4].

While an attractive drug target, the cellular DHODH repertoires of clinically important fungi have not been fully elucidated. Mucorales are predicted to have class 1A DHODH, which would substantiate their lack of olorofim susceptibility [4]. Incidence of mucormycosis has risen due to opportunistic infection caused by COVID-19 and associated treatments [52]. Dematiaceous mould DHODH are not well characterised, but preliminary analyses suggested class 2 enzymes are present in the majority of species [4]. However, olorofim susceptibility is generally lower than *Aspergillus* spp. with some species responding to the drug *in vitro* and some not. These genera can infect immunocompetent individuals, causing chromoblastomycosis (eg. *Fonsecaea*, *Phialophora*.), phaeohyphomycosis (eg. *Alternaria*, *Cladophialophora*, *Exophiala*, *Phialophora*, *Phaeoacremonium*) or eumycetoma (*Madurella*, *Cladophialophora*, *Phialophora*), the latter of which is designated as a Neglected Tropical disease by the World Health Organisation. It is possible that the reduction of olorofim activity in these species is due to the presence of the additional gene products that resemble class 1A DHODH, which may support pyrimidine biosynthesis and cell survival while the class 2 is inhibited. Work published during the course of this study revealed a class 1A DHODH-like gene in *A*. *alternata* to be a dihydrouracil oxidase (DHUO) [53], a poorly characterised type of enzyme described in only one other genus of fungi [54]. DHUO have been seen to convert dihydrouracil (DHU) and dihydrothymine to uracil and thymine, respectively, resembling a step that is the reverse of the initial reaction in the reductive pathway of pyrimidine degradation. The biological context and significance of the conversion of DHU to uracil remains to be elucidated. Deeper understanding of the mechanisms by which fungi regulate pyrimidine levels is needed to inform better drug discovery for fungal targets, in addition to broadening basic knowledge on increasingly problematic pathogens.

While tools are evolving to study gene function in some pathogenic moulds, such as *Rhizopus microsporus* [55] and *R*. *arrhizus* [56], *Mucor lusitanicus* [57] and *E*. *dermatitidis* [58], methods are still lacking for the many species in our study. Further, existing methodologies to knock out genes vary greatly in terms of mechanism, reagents and efficiency. To investigate the functionality of metabolic genes from nine different fungal genera, we opted to utilise the genetically tractable model *Saccharomyces cerevisiae* to obtain reliable and reproducible transformants and results in a time-efficient manner. We set out to understand the DHODH repertoire of clinically important Mucorales and dematiaceous mould species. Bioinformatic analyses of two representative Mucorales species revealed only class 1A DHODH in their proteomes and a yeast complementation study verified these putative proteins could function as DHODH. Biochemical experiments showed these enzymes to be active flavoproteins that use fumarate as electron acceptor, confirming their identity as class 1A DHODH. The same experimental pipeline was applied to seven representative species of dematiaceous fungi. All proteomes assessed appeared to harbour both class 1A-like and class 2-like DHODH; further examination was limited to the putative class 1A-like proteins to understand their importance in the context of olorofim. In contrast to the Mucorales DHODH, complementation analyses and biochemical studies revealed these enzymes to be DHUO, nullifying the hypothesis that these dematiaceous moulds have active class 1A DHODH.

## Materials and methods

### Protein sequence homology analyses

*S*. *cerevisiae* Ura1 and *A*. *fumigatus* PyrE protein sequences were used as class 1A and class 2 DHODH sequences, respectively, to query the proteomes of representative clinically important fungi of interest using BLAST. For *E*. *dermatitidis*, the DHODH sequence from *T*. *cruzi* was used as a query for class 1A orthologs to ensure any class 1A-like sequence was identified in this proteome. Protein sequences were taken from the reference genome for each species. DHODH classification was assigned using Interpro [59]. For phylogenetic analyses, FASTA sequences were submitted to phylogeny.fr (advanced mode) [60]. Sequences were aligned using MUSCLE (full mode) [61] and curated with GBlocks (default settings) [62]. Phylogeny was established by the maximum likelihood method in PhyML, using the approximate likelihood ratio test set to SH-like with WAG substitution [63, 64]. Resultant trees were visualized in TreeDyn [65]. Multiple sequence alignments were performed using MultAlin [66] and visualised graphically using ESPript [67]. All Entrez protein accession numbers used for phylogenetic analysis are listed in **S1 Table**.

### Yeast cloning and plasmid construction

Full-length DHODH genes for heterologous expression in *S*. *cerevisiae* were synthesised in plasmids (Eurofins) or gene blocks (IDT) as native (*T*. *cruzi*, *L*. *kluyveri*, *A*. *fumigatus*, *C*. *bantiana*, *E*. *dermatitidis*, *F*. *pedrosoi*, *P*. *americana*, *A*. *alternata*), *E*. *coli* codon optimised (*R*. *arrhizus*, *M*. *circinelloides*, *A*. *alternata*, *M*. *mycetomatis*, *P*. *minimum*) or *S*. *cerevisiae* codon optimised (*A*. *alternata*) cDNAs with overhangs for downstream cloning. The *A*. *fumigatus* DHODH was cloned from cDNA as described previously [4]. The *S*. *cerevisiae* DHODH gene, was amplified from a BG1805 plasmid containing the URA1 ORF (Horizon Discovery Ltd). All PCR was performed using Phusion HSII polymerase (Thermofisher Scientific). A pYCP shuttle plasmid was designed (VectorBuilder) for heterologous DHODH expression in *S*. *cerevisiae* and contained the following elements: the pUC origin and an ampicillin resistance gene for replication and maintenance in bacteria, the copper-inducible *CUP1* promoter [68], an N-terminal 6xHis tag, the *CYC1* terminator [69], *LEU2* for yeast selection and the CEN6/ARSH4 sequence for replication in yeast [70]. The negative control construct was included in plasmid design and constitutes amino acids 2–83 of *E*. *coli* beta-galactosidase (VectorBuilder). DHODH sequences of interest and controls were inserted behind the N-terminal tag, replacing the negative control sequence, using NEBuilder HiFi (NEB labs). Site-directed mutagenesis was performed either by fusion PCR [71] or using the Phusion Site-Directed Mutagenesis kit (ThermoFisher Scientific).

### Yeast strains, media and genetic methods

The strains used in this study are listed in **S2 Table**. All parental yeast strains were obtained from Horizon Discovery Ltd. The *ura1Δ* strain used in this study was derived from a BY4741 *ura1Δ* strain [72–74] crossed to S288C to make *ura1Δ URA3*^*+*^ *HIS3*^*+*^ strains for transformation with heterologous DHODH plasmids. Genetic crosses were set up on YPD and diploid colonies were selected based on size. Resultant diploids were sporulated in SPO medium (1% KAc, 0.1% Bacto-yeast extract, 0.05% glucose) [75]. Random spore analysis was used to genotype offspring and colony PCR (DreamTaq Green, ThermoScientific) used to confirm the nature of unmarked loci. Yeast strains for transformation were cultured in rich medium with 2% glucose (YPD) and were transformed as described previously [76]. Transformants were selected for on synthetic 2% glucose medium (SD) supplemented with amino acids and vitamins (Formedium) excluding leucine (SD-leu). Serial dilution assays were performed using log-phase cultures grown in SD-leu with 50 μM CuSO_4_ for primary induction of gene expression. Cells were diluted to 1x10^5^ cells/ml and serially diluted tenfold onto appropriate agar plates. Plates were incubated for three days at 30°C. SD-leu lacking uracil (SD-leu-ura) was used to assess the ability of cells to synthesise pyrimidines. Where appropriate DHU was added to plates at a concentration of 150 mg/L. All pYCP-containing strains were grown, assayed, maintained and stored in the absence of leucine to maintain plasmid selection. Anaerobic growth conditions were generated by sealing agar plates in an airtight bag using the ANAEROGEN^TM^ COMPACT system according to the manufacturer’s instructions (Oxoid).

### Olorofim susceptibility testing

To determine the sensitivity of class 1A DHODH and DHUO to olorofim, heterologous yeast strains were subjected to antifungal susceptibility testing according to a modified EUCAST microdilution broth methodology for yeast. Olorofim was tested a concentration range of 0.008–8 mg/L. SD-leu-ura with 1 mM CuSO_4_ broth was used to maintain plasmid selection and ensure pyrimidine synthesis was essential and to ensure robust enzyme expression. For the DHUO strains, 150 mg/L DHU was also added to the broth. Microdilution plates were inoculated with 1x10^6^ cfu/ml and incubated at 35°C for 24 hours. The minimum inhibitory concentration (MIC) was determined visually for each strain. Susceptibility testing was performed in triplicate and results presented as the geometric mean (GM) and range of MICs.

### Bacterial cloning and plasmid construction

To generate protein expression plasmids, full-length cDNAs were cloned into the SmaI and HindIII sites (*S*. *cerevisiae*, *L*. *kluyveri*, *M*. *circinelloides*, *A*. *alternata*, *P*. *minimum*) or SmaI and EcoRI sites (*T*. *cruzi*, *R*. *arrhizus*) of pET50b (Merck) from gene synthesis plasmids (Eurofins) or gene blocks (IDT). Genes were synthesis as native (*T*. *cruzi*, *S*. *cerevisiae*, *L*. *kluyveri*) or *E*. *coli* codon-optimised (all other constructs) cDNAs with flanking restriction sites for cut and paste restriction cloning or overlaps for NEBuilder HiFi (New England Biolabs) assembly into pET50b(+) (Merck). N-terminally truncated *A*. *fumigatus* DHODH (residues 89–531) in pET44 vector (previously described in [19]) was used as a template for PCR by Phusion HSII polymerase (ThermoFisher Scientific). The truncated *A*. *fumigatus* DHODH PCR product was digested with Cfr9I and HindIII (ThermoFisher Scientific) and subcloned into Cfr9I- and HindIII-digested pET50b.

### Recombinant protein expression and purification

Recombinant protein expression was performed as described previously [4]. Briefly, protein expression pET50b plasmids were transformed into *E*. *coli* BL21 (DE3) cells. Heterologous protein expression was induced with 0.5 mM isopropyl β-D-1-thiogalactopyranoside (IPTG) in LB broth in the presence of 30 μg/mL kanamycin and 100 μM flavin mononucleotide (FMN) at 18°C for 16–18 hours. Bacterial pellets were lysed with BugBuster (Merck) and the His-tagged fusion proteins were isolated by immobilized metal affinity chromatography using His-Bind nickel resin (Merck) according to the manufacturer’s instructions. Fusion tags were removed by HRV-3C protease (Merck) by on-column digestion (*M*. *circinelloides*, *A*. *alternata*) or digestion in solution following imidazole elution and desalting (Zeba-spin columns; ThermoFisher Scientific) (all other constructs). Protein concentration was determined by Bradford assay using a Coomassie protein assay kit (ThermoFisher Scientific). 0.75 μg of the final protein fractions were analysed using NuPAGE 4–12% Bis-Tris denaturing gels (Invitrogen) and visualised by staining with GelCode Blue protein stain (ThermoFisher Scientific).

### Flavin binding analysis

The amount of flavin bound to the proteins was determined by fluorescence spectroscopy. Protein samples underwent denaturation for 10 minutes at 99°C followed by cooling on ice and centrifugation at 16,000 g for 2 minutes. The resulting supernatants were analysed using an excitation wavelength of 465 nm and an emission wavelength of 518 nm. The signal of the samples was compared to an FMN standard curve for estimation of flavin bound to the enzymes. As Class 1A DHODHs are known to bind FMN and not FAD, and the fluorescence of FMN is approximately 10-fold that of FAD at pH 8, the flavin was assumed to be FMN.

### Enzyme assays

All enzyme assays were conducted in the following conditions: room temperature, 150 mM KCl, 50 mM Tris-HCl pH 8 buffer, 50 μl reaction volume, 384-well microplates. Enzyme activity was followed spectrophotometrically. DHODH activity was investigated in three different assays, with 1 mM DHO as a substrate. Firstly, 100 μM 2,6-dichloroindophenol (DCIP) was used as a redox indicator whereby oxidation of DHO was coupled to reduction of DCIP that was measured by a decrease in absorbance at 600 nm. 0.1 μg of each protein fraction was added to start the reaction. Secondly, 1 mM potassium hexacyanoferrate III (**f**erricyanide, FeCy) was substituted for the DCIP and reduction of FeCy was followed at 420 nm using 0.05 μg of each protein fraction. Thirdly, orotate production was monitored directly by absorbance at 280 nm in a reaction that included 2 mM sodium fumarate and 0.2 μg protein. DHUO activity was investigated using two assays, with 1 mM DHU as a substrate. Firstly, the production of uracil was followed directly by the increase in absorbance at 260 nm. Secondly, the other product of the reaction, hydrogen peroxide (H_2_O_2_) production was measured by following the increase in absorbance at 595 nm using the Pierce Quantitative Peroxide Assay Kit (ThermoFisher Scientific). 0.2 μg of protein was added to start the reaction for both DHUO assays. For all assays, absorbance results were converted into nanomoles of product produced by the reaction over time or after 30 minutes.

### Olorofim inhibition assays

An adaptation of the DCIP reduction assay described above was used to assess the effect of olorofim and orotate on the activity of DHODH enzymes. The DHO concentration was decreased to 250 μM to allow competition with the product, orotate, which was added at 1 mM where indicated. Olorofim was added at 50 μM where indicated. For the class 2 *A*. *fumigatus* DHODH assay, 0.1% Triton X-100 and 50 μM coenzyme Q2 were included. Reactions (200 μl in 96-well microplates) were monitored at 600 nm to measure DCIP reduction during a 20 minute reaction.

For investigation of the effect of inhibitors on DHUO activity, H_2_O_2_ production was measured as described above except that the DHU concentration was decreased to 250 μM and the reaction time was 20 minutes. Olorofim was added at 50 μM where indicated. Uracil was added at 1 mM where indicated.

### Clustering

DHODH and related sequences from the three domains of life (eukaryotes, bacteria and archaea) were obtained by separate PSI-BLAST searches (three iterations each) using the Tuebingen Toolkit [77]. Redundancy was reduced by using databases non-redundant at 70% sequence identity, and further filtered using MMseqs2 to achieve both minimum sequence identity and minimum alignment coverage of 60%. The results 116 sequences were supplemented with 43 sequences from **S4 Table** and six bacterial proteins related to *Haemophilus paracuniculus* DHODH (NCBI Accession WP_078236156.1). These were submitted to CLANS with default settings [78] and clustered to completion (>100,000 rounds in 2D), excluding outliers that have no BLAST hits to the main group stronger than an E-value of 10^−30^.

## Results

### Mucorales harbour putative class 1A DHODH genes

**Fig 1:**
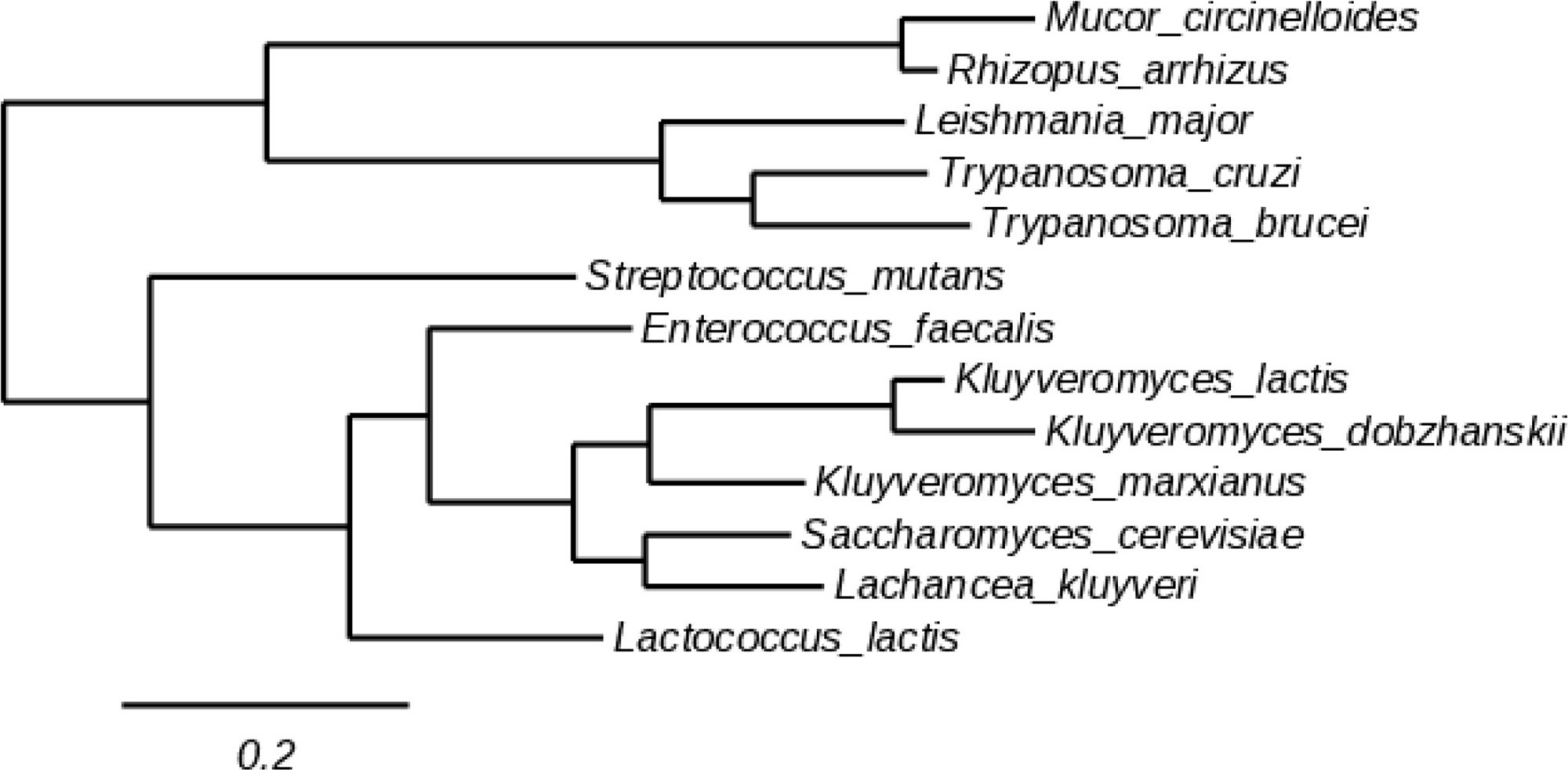
Phylogeny of Mucorales class 1A DHODH. Phylogenetic tree of DHODH sequences from class 1A-containing species. Scale bar is branch length for an expected number of 0.2 substitution per site.

A variety of species are responsible for mucormycosis but *Rhizopus* and *Mucor* spp. cause over 50% of cases [79]. Preliminary phylogenetic analyses have previously suggested that the Mucorales fungi *Rhizopus arrhizus* and *Mucor circinelloides* possess class 1A DHODH [4]. To confirm this, their proteomes were queried with the *S*. *cerevisiae* class 1A (Ura1) and *A*. *fumigatus* class 2 (PyrE) protein sequences using BLASTP (NCBI) (**S1 Table**). Both Mucorales proteomes returned class 1A DHODH proteins with high query coverage and specificity and good identity to the characterised Ura1 protein, indicative of strong homology (**S3 Table**). Further, analysis of these putative sequences using Interpro classified them as belonging to the class 1A family of DHODH based on conserved domains and important sites [59]. No proteins with class 2 homology were detected for either species. Interestingly, phylogenetic analysis showed these putative class 1A grouped with orthologs from *L*. *major* and *Trypanosoma* species rather than yeasts and bacteria (**Fig 1**), concordant with the relatively large phylogenetic distance between Mucormycotina and Saccharomycotina within the fungal kingdom [80]. This analysis suggests that Mucorales species possess only a class 1A DHODH and no class 2 counterpart.

### *Rhizopus* and *Mucor* putative class 1A DHODH genes complement pyrimidine auxotrophy in yeast

**Fig 2:**
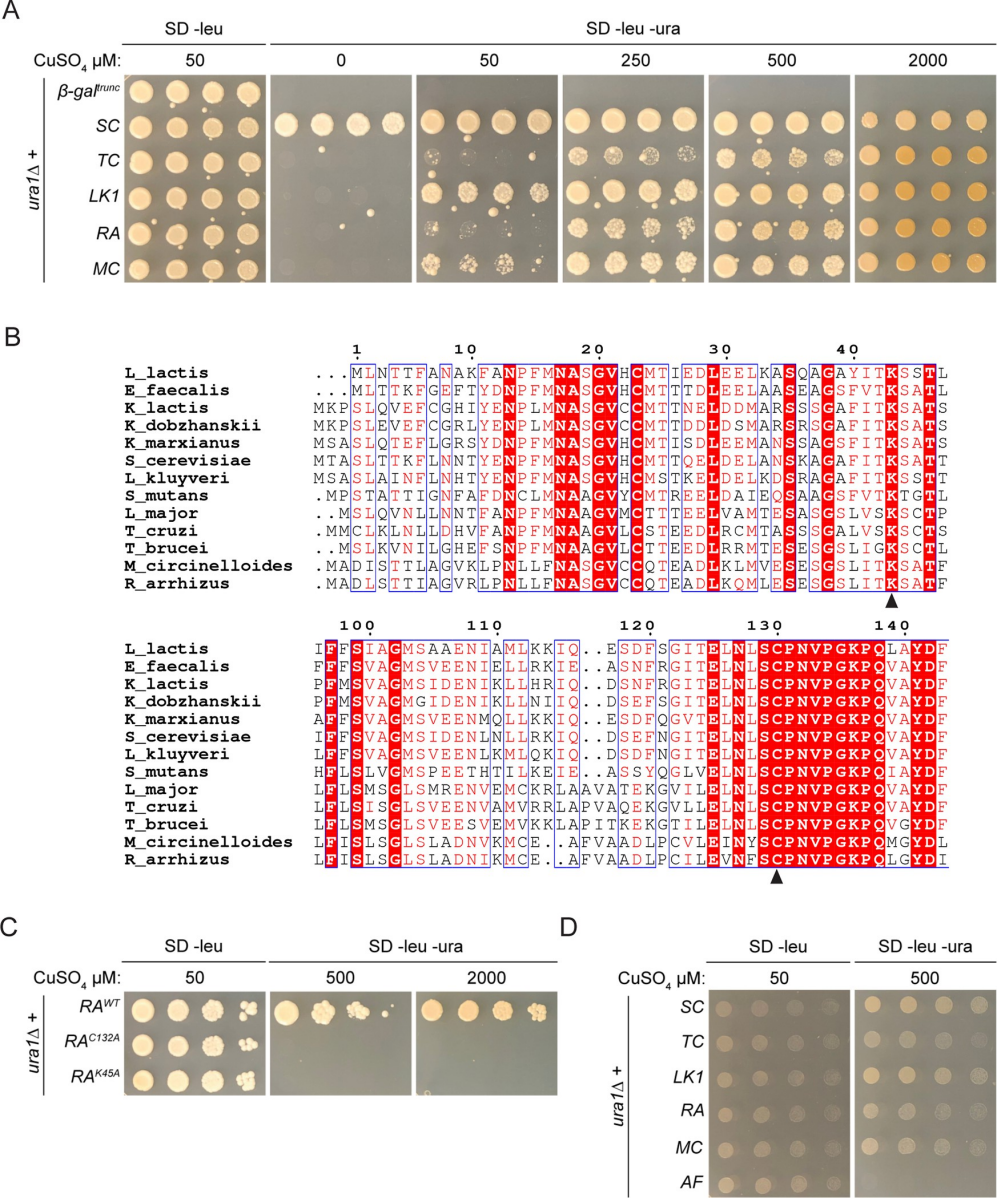
Putative Mucorales class 1A DHODH support pyrimidine prototrophy in yeast. (A) Serial dilution assay of the negative control (β-gal^trunc^) and confirmed class 1A DHODH from *S*. *cerevisiae* (SC), *T*. *cruzi* (TC), *L*. *kluveryi* (LK1A), *R*. *arrhizus* (RA) and *M*. *circinelloides* (MC) complementing *ura1Δ*. (B) Sequence alignment of confirmed class 1A DHODH. Sequences were aligned using MULTALIN [66] and displayed graphically using ESPript [86]. Alignment truncated to show only N-terminus (top) and active site area (bottom). Residue numbers correspond to *L*. *lactis*, black arrowheads denote conserved catalytic amino acids mutated. Red highlight, strict identity; red character, similarity within aligned residues; blue frame, similarity across multiple aligned residues. (C) Serial dilution assay comparing the ability of wild-type (RA^WT^), inactive (RA^C132A^) and lysine mutant (RA^K45A^) *R*. *arrhizus* DHODH to complement *ura1Δ* cells. (D) Serial dilution assay of the confirmed class 1A DHODH from *S*. *cerevisiae* (SC), *T*. *cruzi* (TC), *L*. *kluveryi* (LK1A), *R*. *arrhizus* (RA), *M*. *circinelloides* (MC) and negative control *A*. *fumigatus* (AF) complementing *ura1Δ* in anaerobic conditions.

To verify the putative proteins identified bioinformatically are class 1A DHODH, their activity was first assessed using a heterologous system. Previous studies demonstrated that both fungal [43, 44] and non-fungal [81] DHODH can complement *ura1Δ S*. *cerevisiae* strains. Therefore, a *ura1Δ* system was utilised to screen the activity of the putative enzymes through complementation. DHODH sequences and controls were inserted into a vector behind the *CUP1* promoter that has leaky expression in regular medium but expression increases with increasing concentrations of Cu^2+^ [68]. A truncated *E*. *coli* gene (beta-galactosidase) and *S*. *cerevisiae URA1*^*+*^ were included as a negative and positive controls, respectively. Further, characterised class 1A genes from other species were also used to ensure robustness of the system: the yeast *L*. *kluyveri*, which also has a functional class 2 DHODH, and the parasite *T*. *cruzi*. To ensure an overabundance of copper is not toxic to yeast cells, active class 1A DHODH were induced with a high concentration of copper in pyrimidine-rich media and viability assessed (**S1A Fig**). Indeed, *ura1Δ* strains expressing either *S*. *cerevisiae*, *T*. *cruzi* or *L*. *kluyveri* class 1A DHODH (SC, TC, LK1A, respectively) were viable in medium containing 2000 μM copper and uracil, implying no toxic effects of copper accumulation. In line with previous observations, cells were slightly darker in colour due to accumulation of copper sulfide [82, 83]. Transformants were then assessed for their ability to grow in the absence of pyrimidines in various inducing conditions, indicative of DHODH activity (**Fig 2A**). As expected, the negative control was unable to complement in any condition while SC supported robust growth with just leaky basal expression. LK1A was able to rescue growth in mildly inducing conditions (50 μM CuSO_4_) and even the non-fungal TC produced robust growth, albeit at very high concentrations of copper (2000 μM CuSO_4_), reflective of its evolutionary distance. Interestingly, both Mucorales DHODH functioned in yeast but required slightly higher levels of induction for complete pyrimidine prototrophy, compared to the two other fungal enzymes.

To confirm that DHODH activity was specifically responsible for the rescue of *ura1Δ*, predicted key catalytic residues were mutated in the *R*. *arrhizus* gene and complementation assessed. The active site cysteine and an N-terminal lysine involved in catalysis were identified in *R*. *arrhizus* [84, 85] (**Fig 2B**) and both were mutated to alanine to negate their function. Both RA^C132A^ and RA^K45A^ were unable to restore growth in the absence of pyrimidines in any concentration of copper (**Fig 2C**), confirming that production of orotate by class 1A DHODH is responsible for complementation.

Class 1A DHODHs support anaerobic growth through independence of pyrimidine biosynthesis from the respiratory chain and facilitate facultative anaerobiosis in *S*. *cerevisiae* and *L*. *kluyveri* [25, 42, 43]. To understand if the *Mucorales* DHODH shared this property, *ura1Δ* rescue was assessed under anaerobic conditions. Some class 2 DHODH do not support anaerobic pyrimidine prototrophy in this yeast system [43, 44], therefore the DHODH from *A*. *fumigatus* was included as a negative control as this enzyme is not toxic and rescues *ura1Δ* in aerobic conditions (**S1B Fig**). Growth was slower than that of aerobic conditions but all class 1A DHODH rescued growth under anaerobic conditions, in sharp contrast to the *A*. *fumigatus* class 2 (**Fig 2D**). In this robust system, Mucorales putative class 1A genes appear to have DHODH activity.

### *Rhizopus* and *Mucor* enzymes have class 1A DHODH activity *in vitro*

**Fig 3:**
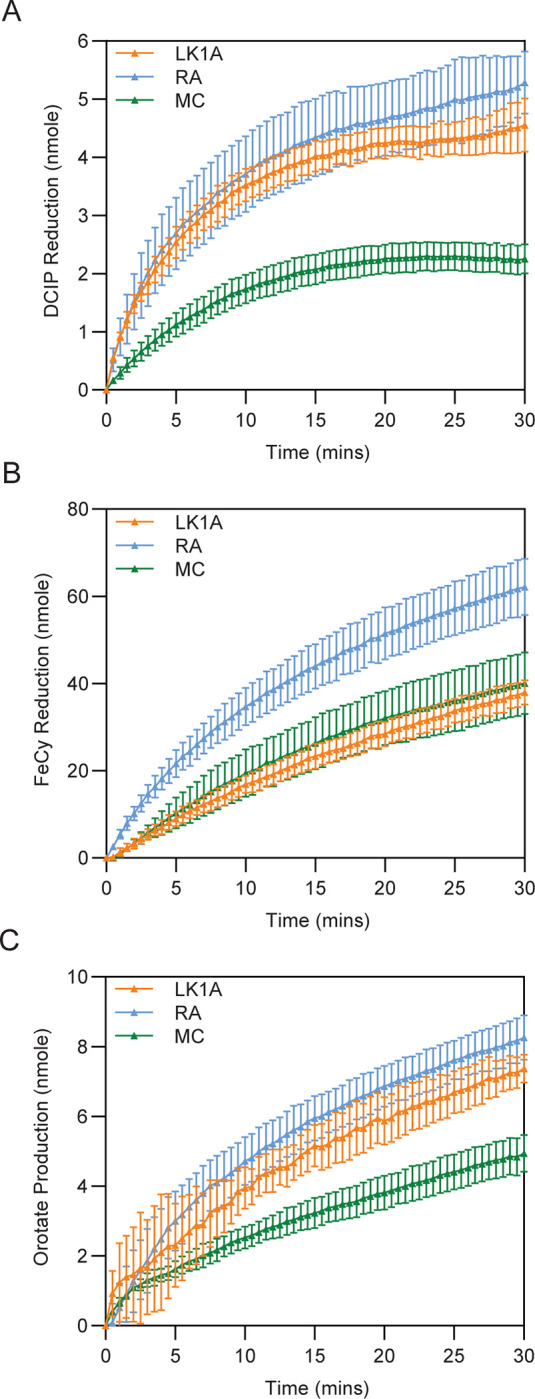
Mucorales class 1A DHODH are active *in vitro*. Biochemical analyses of *L*. *kluyveri* class 1A (LK1A), *R*. *arrhizus* (RA) and *M*. *circinelloides* (MC) DHODH. (A) Reduction of DCIP by DHODH over time. (B) Reduction of FeCy by DHODH over time. (C) Production of orotate by DHODH over time.

Class 1A DHODH activity for these two Mucorales species was confirmed further using a biochemical approach. Full-length, codon-optimised forms of *R*. *arrhizus* and *M*. *circinelloides* DHODH cDNAs were synthesised, and recombinant proteins expressed and purified from *E*. *coli* (**S2 Fig**). Initial characterisation of these constructs as DHODH showed they bind flavin in a molar ratio similar to those observed for *S*. *cerevisiae*, *L*. *kluyveri* 1A and *T*. *cruzi* DHODH and to previous reports for class 1A DHODH [87] indicating they are flavoproteins (**Table 2**). The similarity in flavin fluorescence among the Mucorales and classified class 1A DHODH indicates that it is FMN and not FAD bound to these proteins. Like other characterised DHODHs, both Mucorales enzymes could reduce two artificial electron acceptors: 2,6-dichloroindophenol (DCIP) (**Fig 3A**) and potassium hexacyanoferrate III (ferricyanide, FeCy) (**Fig 3B**) in the presence of DHO. Moreover, both enzymes could convert DHO to orotate in the presence of the native class 1A electron acceptor fumarate (**Fig 3C**). The *R*. *arrhizus* enzyme appeared to have higher activity than the *M*. *circinelloides* enzyme in all three assays. Taken together, these data suggest that these Mucorales species have active class 1A DHODH enzymes.

**Table 2:** FMN binding of recombinant class 1A DHODH. Ratio of FMN to protein of *L*. *kluyveri* class 1A (LK1A), *T*. *cruzi* (TC), *R*. *arrhizus* (RA) and *M*. *circinelloides* (MC) DHODH.

DHODH	SC	LK1A	TC	MC	RA
**μmole FMN/μmole protein**	0.64	0.68	0.79	1.06	0.73

### Class 1A DHODH are not inhibited by olorofim

With the DHODH repertoire of the Mucorales species characterised, we set out to confirm that these enzymes are not inhibited by olorofim and as such are responsible for the resistance of these species. Firstly, the class 1A DHODH heterologous yeast strains were subjected to olorofim susceptibility testing using a broth microdilution method and the minimum inhibitory concentration (MIC) of olorofim determined for each strain. Wild-type was used as a negative control for olorofim activity and the strain expressing the olorofim-susceptible *A*. *fumigatus* class 2 DHODH was used as a positive control. As expected, growth of the heterologous *A*. *fumigatus* strain was strongly inhibited with a mean MIC of 0.125 mg/L (**Table 3**), reflective of the potency of olorofim for this DHODH and confirms olorofim could permeate *S*. *cerevisiae* cells [4]. The wild-type strain had an MIC of >8 mg/L, suggesting endogenous Ura1 is not inhibited by this antifungal nor are there any off-target effects in this species. All class 1A DHODH strain growth was completely unaffected, suggesting olorofim cannot inhibit class 1A DHODH activity in the yeast system.

**Table 3:** Olorofim sensitivity of heterologous yeast strains expressing class 1A DHODH. The geometric mean (GM) MIC and MIC range are presented for a wild-type (*WT*) strain and heterologous strains expressing either the class 2 DHODH from *A*. *fumigatus (AF)* or the class 1A DHODH from *S*. *cerevisiae (SC)*, *T*. *cruzi (TC*), *L*. *kluyveri (LK1A)*, *M*. *circinelloides (MC)* and *R*. *arrhizus (RA)*.

		*ura1Δ* +					
	*WT*	*AF*	*SC*	*TC*	*LK1A*	*MC*	*RA*
**GM (mg/L)**	>8	0.125	>8	>8	>8	>8	>8
**Range (mg/L)**	>8	0.125	>8	>8	>8	>8	>8

**Fig 4:**
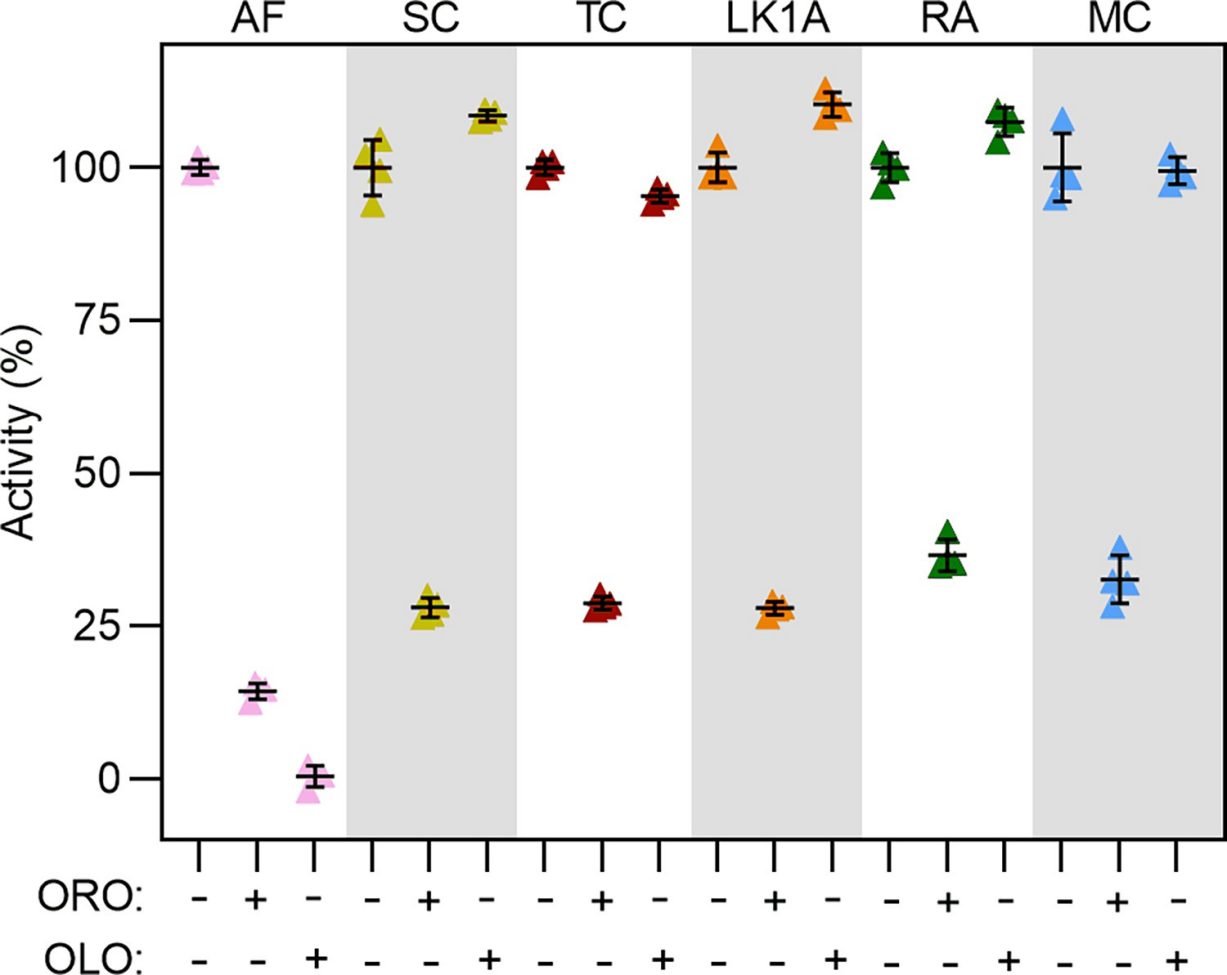
Class 1A DHODH are not inhibited by olorofim. DCIP reduction assays of *A*. *fumigatus* (AF), *S*. *cerevisiae* (SC), *T*. *cruzi* (TC), *L*. *kluveryi* (LK1A), *R*. *arrhizus* (RA) and *M*. *circinelloides* (MC) in the absence (-) or presence (+) of either orotate (ORO) or olorofim (OLO). Activity in the presence of inhibitors was expressed as a percentage of control enzyme activity in absence of inhibitors after 20 minutes (100%).

To directly address whether olorofim has any effect on these class 1A DHODH, inhibition assays were performed using the purified proteins and DCIP reduction was monitored. Recombinant class 2 *A*. *fumigatus* DHODH was used as a positive control for olorofim activity [4]. The catalytic product orotate was used as a control for general DHODH inhibition, demonstrating >60% inhibition of activity against all enzymes (<50% activity) (**Fig 4**). While *A*. *fumigatus* enzyme activity dropped to almost zero in the presence of olorofim, the antifungal had no inhibitory effect for any of the class 1A DHODH tested, indicative of its selectivity for class 2 enzymes.

### Dematiaceous moulds appear to harbour two classes of putative DHODH genes

**Fig 5:**
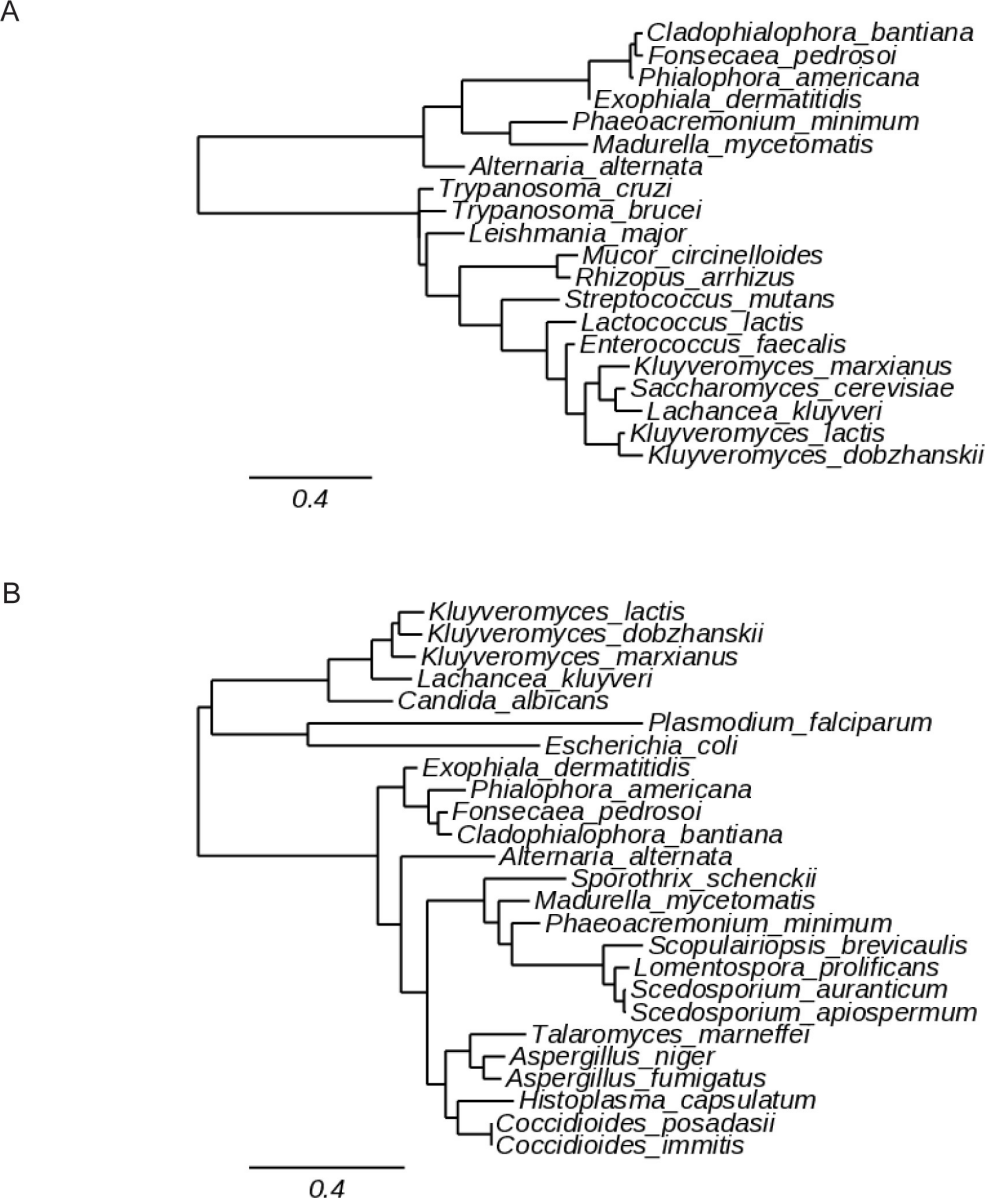
Phylogeny of dematiaceous mould DHODH. Phylogenetic trees of (A) class 1A and (B) class 2 DHODH sequences. Scale bars represent branch length for an expected number of 0.4 substitution per site.

To determine the class of DHODH present in the dematiaceous moulds, their proteomes were queried for class 1A and class 2 orthologs, again using Ura1 and PyrE, respectively. All seven species returned both putative class 1A DHODH- and class 2 DHODH-like proteins with varying degrees of sequence identity to the queries, however sequence identity and E-values were consistently better for PyrE (>54% identity) than for Ura1 (24–31%) in all species (**S4 Table**). However, Interpro classified all Ura1 orthologs as belonging to the class 1A DHODH protein family. Remarkably, class 1A DHODH sequences from a range of species revealed the dematiaceous moulds form a distinctly separate clade to the fungi proven to have the enzyme (**Fig 5A**) The dematiaceous class 2 sequences are slightly more integrated with other species containing this type of enzyme, suggesting these enzymes are more closely related than the putative class 1A are (**Fig 5B**). This analysis suggests dematiaceous moulds may have both class 1A and class 2 DHODH. We investigated these phylogenetically distinct putative class 1A proteins further to determine whether they had DHODH activity.

### Dematiaceous mould putative class 1A DHODH genes do not complement pyrimidine auxotrophy in yeast

**Fig 6:**
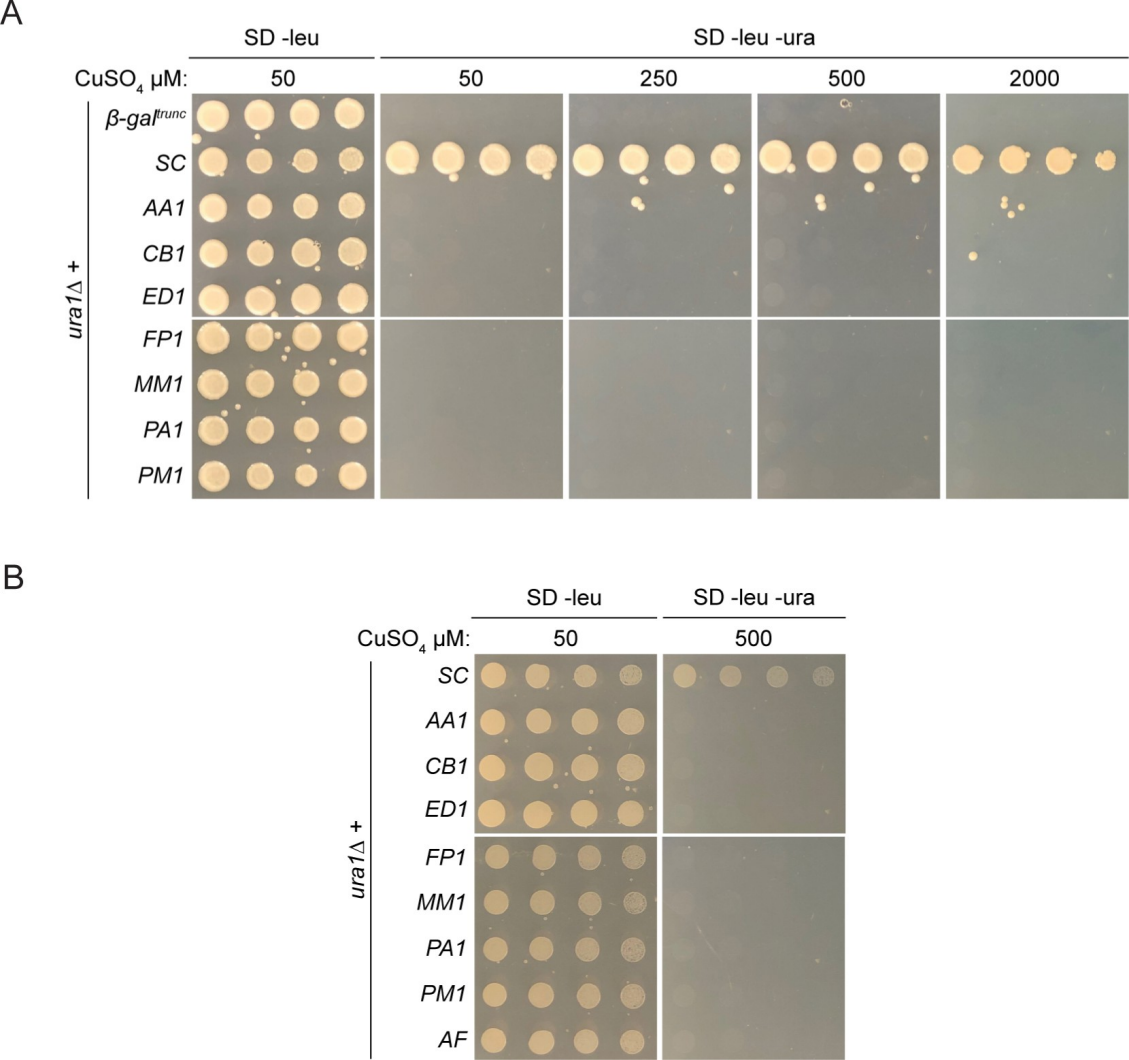
Dematiaceous mould genes do not support pyrimidine prototrophy in yeast. (A) Serial dilution assay of the negative control (β-gal^trunc^), confirmed class 1A DHODH from *S*. *cerevisiae* (SC) and putative genes from *A*. *alternata* (AA), *C*. *bantiana* (CB), *E*. *dermatitidis* (ED), *F*. *pedrosoi* (FP), *M*. *mycetomatis* (MM), *P*. *americana* (PM) and *P*. *minimum* (PM) in *ura1Δ* cells (B) Serial dilution assay of the negative control (β-gal^trunc^), confirmed class 1A DHODH from *S*. *cerevisiae* (SC) and putative class 1A DHODH from *A*. *alternata* (AA), *C*. *bantiana* (CB), *E*. *dermatitidis* (ED), *F*. *pedrosoi* (FP), *M*. *mycetomatis* (MM), *P*. *americana* (PM) and *P*. *minimum* (PM) in *ura1Δ* cells under anaerobic conditions.

With evidence that heterologous class 1A activity from species with multiple DHODH genes produces sufficient pyrimidines for *ura1Δ* growth, the activity of the putative class 1A DHODH genes from dematiaceous moulds was assessed using the same system. In sharp contrast to SC, class 1A constructs from all seven species evaluated were unable to complement *ura1Δ*, even under strong inducing conditions (2000 μM CuSO_4_) (**Fig 6A**).

To ensure that codon usage was not a limiting factor in the ability of these exogenous genes to rescue *ura1Δ*, three variations of the *A*. *alternata* class 1A were assessed for complementation. An *E*. *coli* codon-optimised construct (AA1A^Ec^), a native CDS construct (AA1A^Nat^) and a yeast-codon optimised construct (AA1A^Sc^) all behaved similarly in complementation testing; none rescued the *ura1Δ* strain (**S3 Fig**). Therefore, any potential translation issues are not responsible for lack of DHODH activity.

In attempts to uncover any potential DHODH functionality of these putative genes, their activity was investigated under anaerobic conditions. Only *ura1Δ* strains expressing confirmed class 1A DHODH were able to grow anaerobically. Like the *A*. *fumigatus* DHODH, the putative enzymes were unable to support anaerobic growth (**Fig 6B**). Taken together, these results suggest that these putative class 1A genes do not harbour DHODH activity.

### Dematiaceous mould putative genes use DHU to support yeast pyrimidine prototrophy

**Fig 7:**
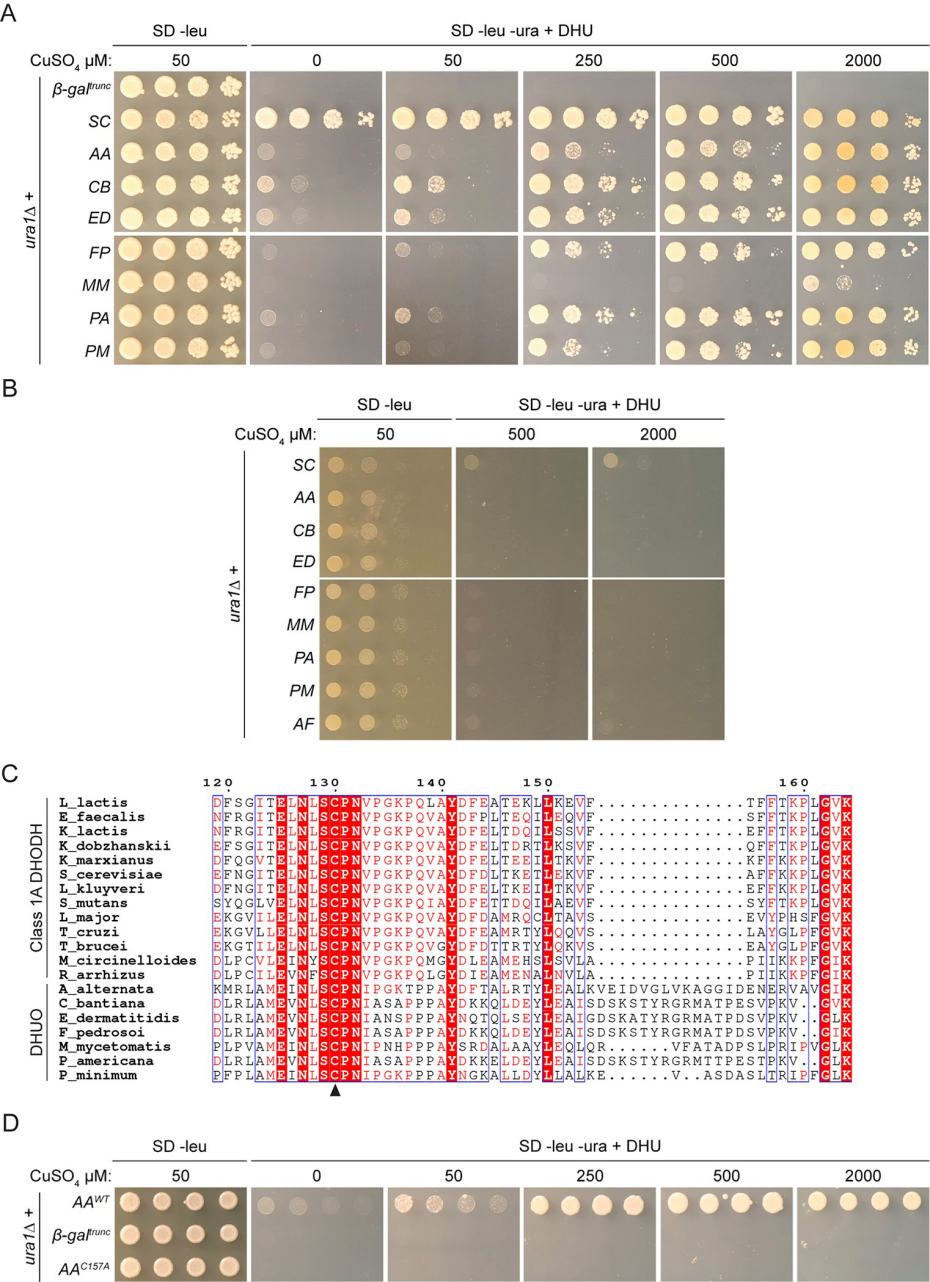
Dematiaceous mould putative genes have DHUO activity in the yeast model. (A) Serial dilution assay of the negative control (β-gal^trunc^), confirmed class 1A DHODH from *S*. *cerevisiae* (SC) and putative genes from *A*. *alternata* (AA), *C*. *bantiana* (CB), *E*. *dermatitidis* (ED), *F*. *pedrosoi* (FP), *M*. *mycetomatis* (MM), *P*. *americana* (PM) and *P*. *minimum* (PM) in *ura1Δ* cells in the presence of DHU. (B) Serial dilution assay of the confirmed class 1A DHODH from *S*. *cerevisiae* (SC) and putative genes from *A*. *alternata* (AA), *C*. *bantiana* (CB), *E*. *dermatitidis* (ED), *F*. *pedrosoi* (FP), *M*. *mycetomatis* (MM), *P*. *americana* (PM) and *P*. *minimum* (PM) in *ura1Δ* cells under anaerobic conditions in the presence of DHU. (C) Sequence alignment of confirmed class 1A DHODH and dematiaceous DHUO. Sequences were aligned using MULTALIN [66] and displayed graphically using ESPript [86]. Alignment truncated to show only active site area. Residue numbers correspond to *L*. *lactis*, black arrowhead denotes conserved catalytic cysteine mutated. Red highlight, strict identity; red character, similarity within aligned residues; blue frame, similarity across multiple aligned residues. (D) Serial dilution assay comparing the ability of wild-type (AA^WT^), negative control (β-gal^trunc^) and cysteine mutant (AA^C157A^) *A*. *alternata* DHUO to complement *ura1Δ* cells.

During the course of this study, another group demonstrated that yeast cell lysates containing the *A*. *alternata* putative class 1A DHODH gene could oxidise DHU to uracil and as such the enzyme has been classified as a DHUO [53]. In the absence of DHODH activity and considering the *A*. *alternata* findings, the other dematiaceous moulds were assessed for DHUO activity in the yeast complementation system. Transformants were again assayed for their ability to grow in the absence of pyrimidines in various inducing conditions but in the presence of DHU [53], indicative of their ability to oxidise the substrate to uracil and rescue the *ura1Δ* pyrimidine auxotrophy. As expected, the negative control was unable to complement in any condition and the SC growth control grew well in all conditions (**Fig 7A**). The previously characterised *A*. *alternata* DHUO [53] rescued the auxotrophy well at 250 μM copper and above. Intriguingly, all dematiaceous enzymes complemented *ura1Δ* growth using DHU, implicating them as DHUO, but pyrimidine prototrophy was not identical for each DHUO at equivalent copper levels, suggestive of variation in oxidase activity between enzymes of different species. In line with previous findings [53], none of the dematiaceous mould enzymes could use DHU to rescue pyrimidine auxotrophy in anaerobic conditions (**Fig 7B**). These data show that these putative enzymes use DHU and oxygen to produce uracil, implicating them as DHUO rather than DHODH. Again, codon usage had no effect on ability of a construct to rescue *ura1Δ* the in the presence of DHU (**S4 Fig**).

To confirm that DHUO activity was specifically responsible for the rescue of *ura1Δ*, the predicted key catalytic residue was identified and mutated in the *A*. *alternata* gene and complementation assessed. The potential active was identified in the dematiaceous fungi through sequence alignment with confirmed class 1A DHODH, where a clear SCPNV motif was conserved across both enzyme classes (**Fig 7C**). On the hypothesis that class 1A DHODH and DHUO catalytic mechanisms may share an important cysteine, this residue was mutated to alanine in *A*. *alternata* to negate activity. AA^C157A^ was unable to restore growth using DHU in the absence of pyrimidines in any concentration of copper (**Fig 7D**). These results not only confirm that production of uracil is responsible for complementation in this system but provides the first insights into the sequence and catalytic mechanism of DHUO enzymes.

### Dematiaceous mould enzymes display DHUO activity *in vitro*

**Fig 8:**
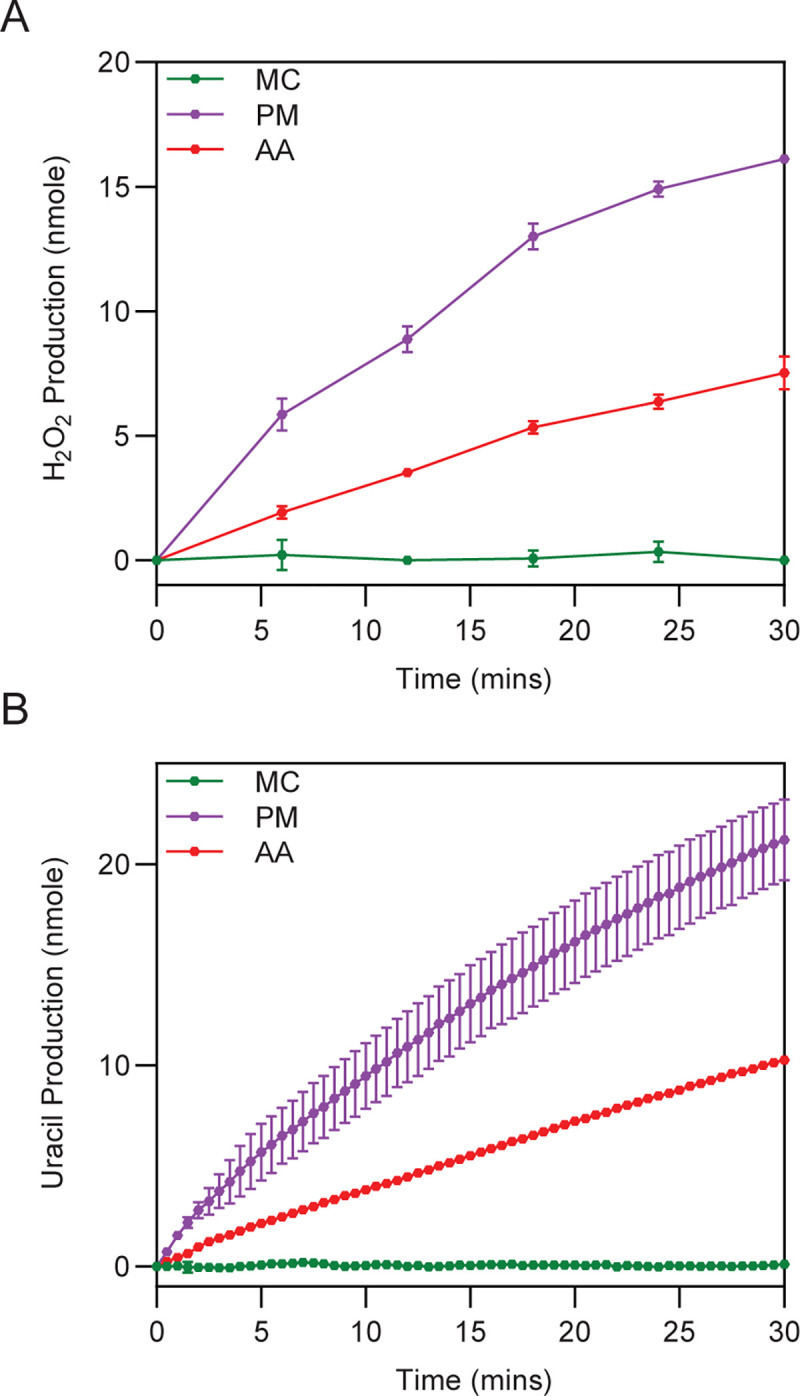
Dematiaceous mould enzymes have DHUO activity *in vitro*. Biochemical analyses of *M*. *circinelloides* class 1A DHODH (MC) and *A*. *alternata* (AA) and *P*. *minimum* (PM) DHUO. (A) Production of uracil over time. (B) Production of H_2_O_2_ over time.

Enzymatic identity and activity for representative dematiaceous proteins were further assessed biochemically. Full-length, codon-optimised *A*. *alternata* and *P*. *minimum* cDNAs were synthesised and recombinant protein expressed and purified from *E*. *coli* (**S5A Fig**). At 37.5 and 35.5 kDa, respectively, these proteins are slightly bigger than the five class 1A DHODHs studied above, ranging from 33.6 to 35.1 kDa. Again, characterisation of these enzymes as flavoproteins revealed them to bind flavin with a molar ratio similar to that observed for the known FMN-binding class 1A DHODHs, indicating that it is FMN and not FAD bound (**Table 4**). The observed lack of DHODH activity observed in the complementation study was confirmed by following the reduction of DCIP and FeCy and production of orotate in the presence of DHO; neither dematiaceous enzyme was active in any of these *in vitro* assays compared to class 1A DHODHs (**S5B–S5D Fig**), supporting the notion that these enzymes are not class 1A DHODH. Subsequently, based on findings from Bouwknegt and colleagues, DHUO activity for these enzymes was assessed *in vitro*, following production of uracil and hydrogen peroxide levels as the two products of DHU oxidation [54, 88]. Concordant with previous observations, the *A*. *alternata* enzyme as well as that from *P*. *minimum* converted DHU to uracil while the *M*. *circinelloides* class 1A DHODH could not (**Fig 8A**). Furthermore, both dematiaceous enzymes produced hydrogen peroxide while the proven class 1A had no such activity (**Fig 8B**). The enzyme from *P*. *minimum* appeared to be more active than the *A*. *alternata* counterpart in both DHUO assays. Taken together, these data suggest that these species of dematiaceous mould have active DHUOs.

**Table 4:** FMN binding of dematiaceous mould enzymes. Ratio of FMN to protein of *A*. *alternata* (AA) and *P*. *minimum* (PM) DHUO.

DHODH	AA	PM
**μmole FMN/μmole protein**	0.50	0.91

### DHUO are not inhibited by olorofim

With the knowledge that these dematiaceous genes are not DHODH, we set out to understand whether olorofim has any effect on this class of enzymes. Like the wild-type strain, all DHUO strain growth was completely unaffected, implying olorofim does not inhibit heterologous DHUO activity (**Table 5**).

**Table 5:** Olorofim sensitivity of heterologous yeast strains expressing DHUO. The geometric mean (GM) MIC and MIC range are presented for a wild-type (*WT*) strain and heterologous strains expressing either the class 2 DHODH from *A*. *fumigatus (AF)* or the DHUO from *A*. *alternata (AA)*, *C*. *bantiana (CB*), *E*. *dermatitidis (ED)*, *F*. *pedrosoi (FP)*, *M*. *mycetomatis (MM)*, *P*. *americana (PA)* and *P*. *minimum (PM)*.

		*ura1Δ* +							
	*WT*	*AF*	*AA*	*CB*	*ED*	*FP*	*MM*	*PA*	*PM*
**GM (mg/L)**	>8	0.125	>8	>8	>8	>8	>8	>8	>8
**Range (mg/L)**	>8	0.125	>8	>8	>8	>8	>8	>8	>8

**Fig 9:**
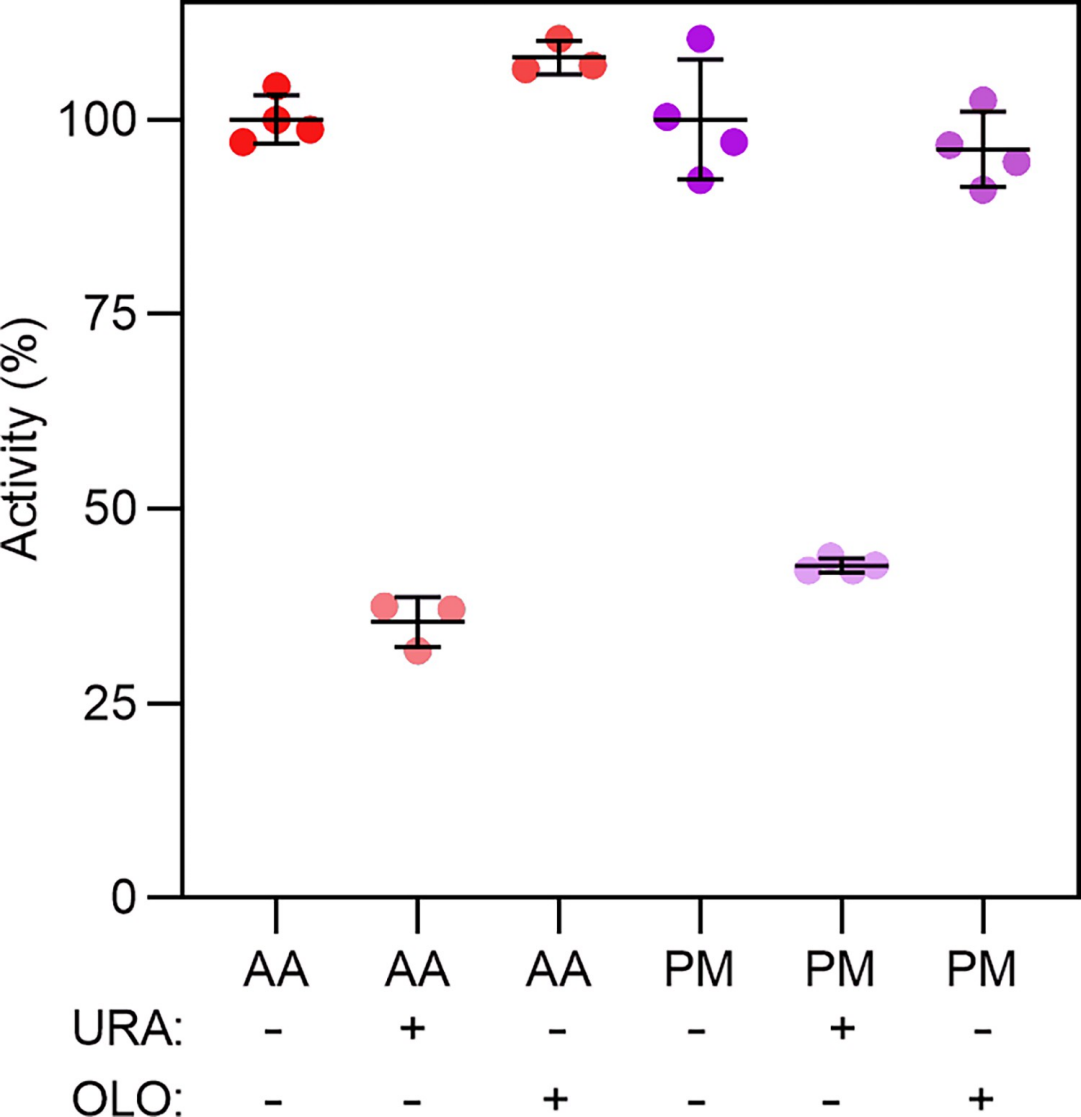
DHUO are not inhibited by olorofim. H_2_O_2_ production assays of *A*. *alternata* (AA) and *P*. *minimum* (PM) DHUO in the absence (-) or presence (+) of either uracil (URA) or olorofim (OLO). Activity in the presence of inhibitors was expressed as a percentage of control enzyme activity in absence of inhibitors after 20 minutes (100%).

Building on the yeast system results, the direct effects of olorofim on DHUO were investigated biochemically using the hydrogen peroxide assay. The catalytic product uracil was used as a positive control for DHUO inhibition [88], reducing both *A*. *alternata* and *P*. *minimum* enzyme activity to less than 50% (**Fig 9**). However, olorofim showed no substantial inhibitory effect for either DHUO, confirming that this antifungal does not target this class of enzymes.

## Discussion

DHODH are ubiquitous enzymes important to cellular metabolism, yet their diversity between and within classes makes them attractive therapeutic targets. In this study, we have shed light on the DHODH repertoire of two different types of clinically important fungi. Firstly, we have validated the prior suggestion that the Mucorales species *M*. *circinelloides* and *R*. *arrhizus* harbour active class 1A DHODH; these enzymes utilise fumarate to produce orotate and are able to functionally replace the *S*. *cerevisiae* class 1A enzyme. Secondly, we have disproved our hypothesis that certain dematiaceous moulds may contain class 1A DHODH: these putative enzymes behave as DHUO that catalyse the oxidation of DHU to uracil *in vitro* and in the yeast system tested.

Preliminary bioinformatics from our previous study suggested Mucorales species harbour class 1A DHODH due to their sequence similarity to known enzymes of this class [4]. Building on this work, proteomes of the Mucorales were found to harbour only orthologs of class 1A DHODH; we pursued characterisation of enzymes from two representative species that are the most common etiological agents of mucormycosis. As with all characterised class 1A DHODHs, the *M*. *circinelloides* and *R*. *arrhizus* enzymes were seen to be flavoproteins that bound FMN with a molar ratio of FMN to protein of 1.06 and 0.73, respectively, values that support the presence of one FMN molecule per subunit in active enzyme. Both enzymes were able to utilise fumarate as a terminal electron acceptor typical of class 1A DHODH for oxidation of DHO to orotate. It is interesting to note that the *R*. *arrhizus* DHODH was more active than the other Mucorales enzyme and even the *L*. *kluyveri* class 1A, perhaps reflective of different nutritional requirements in different species. Moreover, their ability to support anaerobic pyrimidine prototrophy in yeast provides further evidence that these enzymes belong to the cytosolic class that do not interact with the respiratory chain, allowing oxygen-independent growth in some species [43]. *Mucor* spp. can grow anaerobically and exhibit morphological dimorphism between aerobic and anaerobic conditions [89, 90], in line with an oxygen-independent method of synthesising pyrimidines using class 1A DHODHs [25]. We also confirmed that the class 2 DHODH of *A*. *fumigatus* was unable to support anaerobic pyrimidine prototrophy which is consensus for most class 2 DHODHs, as they donate electrons to the respiratory chain [25], but not for all [44]. Further, we expanded on structural observations that an N-terminal lysine is vital for catalysis by making a hydrogen bond to the FMN cofactor [85, 91]: mutagenesis of this residue in the *R*. *arrhizus* enzyme replicated the loss of activity seen in mutation of the active site cysteine responsible for class 1A catalysis. While general sequence similarity is low among class 1A DHODHs, conservation of important residues across kingdoms is useful for future therapeutic drug design and so further mutagenesis studies should be conducted in a variety of species. Characterisation of these Mucorales DHODH as class 1A enzymes substantiates the lack of olorofim activity seen for this group of moulds. In fact, the drug had no indirect or direct activity on any class 1A DHODH tested in yeast or biochemically, respectively. Olorofim susceptibility and the identity and activity of DHODH enzymes in other species of the Mucorales order of fungi, though predictable from this study, remain to be confirmed but would enrich the current understanding of these opportunistic pathogens.

The same prior phylogenetic analyses also found class 2 DHODH in a limited number of dematiaceous moulds, including *Alternaria* spp. and *Exophiala*, although sequences grouped away from those of genera against which olorofim is most potent, such as *Aspergillus* spp., *Coccidioides*, *Histoplasma and Blastomyces* [4]. We posited that the lack of olorofim activity in some of these species that appear to contain the drug target may be due to the presence of a secondary DHODH of a different class, specifically a class 1A as is the repertoire of *L*. *kluyveri* [42, 43]. Indeed, we found DHODH-like proteins belonging to both class 1A and class 2 families in this selection of dematiaceous moulds, though overall the class 2 orthologs had stronger identity to PyrE than the class 1As did to Ura1. Nevertheless, the putative Ura1 orthologs had enough conservation of important residues for them to be classified as class 1A DHODH proteins. The presence of putative class 1A in all species queried was unexpected as some show sensitivity to olorofim, such as *M*. *mycetomatis* and *Phaeoacremonium* spp. [17, 18], while others have lowered susceptibility, including *A*. *alternata* and *E*. *dermatitidis* [9, 19]. With the view to understand the effects of a secondary DHODH in species containing the olorofim target DHODH, we focused on characterising the putative class 1A DHODH genes in a selection of dematiaceous moulds, with remarkable results.

Surprisingly, no putative class 1A DHODH gene from any of the pathogenic species was able to support aerobic or anaerobic pyrimidine prototrophy in *S*. *cerevisiae*. Further, purified recombinant *A*. *alternata* and *P*. *minimum* proteins did not exhibit classical class 1A biochemistry; neither could produce orotate or use any of the electron acceptors tested. However, both enzymes bound FMN, indicating they are flavoproteins but lack apparent DHODH activity. These data were unexpected as annotation of most of these genes and protein products assigned or predicted them to be class 1A DHODH.

**Fig 10:**
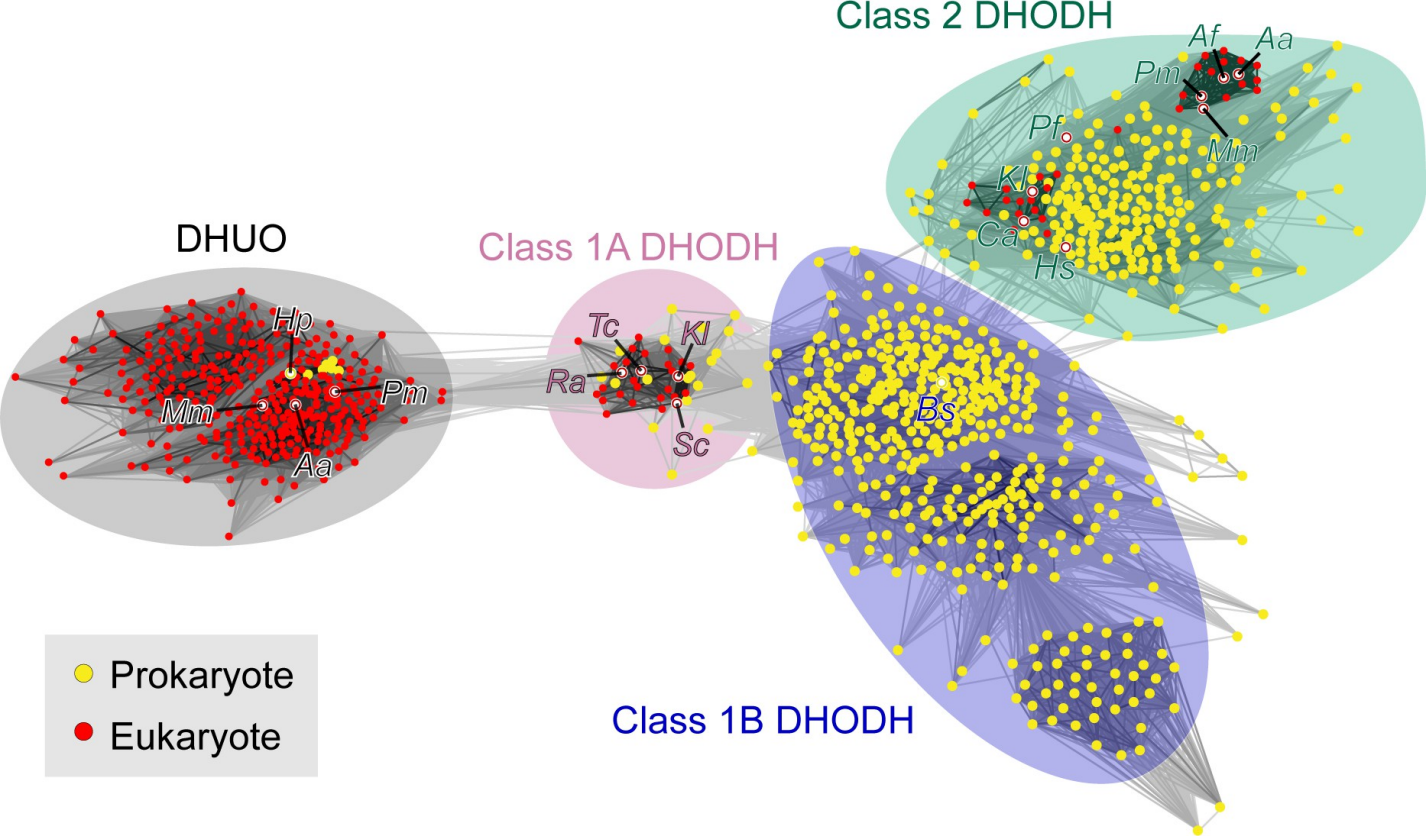
DHUOs are a separate enzyme family in fungi and bacteria. Cluster map of >1000 DHODH sequences obtained from all domains of organisms, indicating the classes of DHODH (1A, 1B and 2) and the origin in eukaryotes (red) or prokaryotes (yellow). A small number of species are highlighted by names: *A*. *alternata (Aa)*, *A*. *fumigatus (Af)*, *B*. *subtilis (Bs)*, *C*. *albicans (Ca)*, *H*. *paracuniculus (Hp)*, *H*. *sapiens (Hs)*, *K*. *lactis (Kl)*, *M*. *mycetomatis (Mm)*, *P*. *falciparum (Pf)*, *P*. *minimum (Pm)*, *R*. *arrhizus (Ra)*, *S*. *cerevisiae (Sc)* and *T*. *cruzi (Tc)*.

While our work was underway, a study used yeast complementation and lysate assays to demonstrate that the Ura1-like gene from *A*. *alternata* encodes a DHUO: a redox enzyme that utilises oxygen to convert DHU or dihydrothymine into uracil or thymine, plus hydrogen peroxide [53]. In line with this work, we subsequently found that the putative dematiaceous enzymes facilitated pyrimidine prototrophy in yeast when supplemented with DHU but only under aerobic conditions. Our biochemical data provide direct evidence that *A*. *alternata* possesses a DHUO and that *P*. *minimum* can be added to the list of organisms with this class of metabolic enzyme. Limited work on DHUO has demonstrated that they are flavoproteins that utilise only oxygen as an electron acceptor for oxidase activity [88]. Our observations corroborate this; both dematiaceous enzymes bind FMN and cannot rescue yeast growth in anaerobic conditions even in the presence of DHU. Expanding on preliminary sequence analysis [53], all class 1A DHODH and DHUO proteins assessed in this study shared a highly conserved active site that is likely a large contribution to the *in silico* assignment of the latter as the former in protein databases. Further, we provide the first insight into the mechanism of DHU oxidation through the identification of a catalytic cysteine in the *A*. *alternata* enzyme. Conservation of this cysteine across DHUO and class 1A DHODH is remarkable yet unsurprising as sequence or structure is frequently seen to be similar among enzymes that catalyse different reactions as an example of divergent evolution [92], providing an insight into a possible relationship between these two classes of metabolic enzyme. Assessing sequence similarity between enzymes from this study and across all of evolution defines the DHUOs as a separate, large family of enzymes that group together away from the three well-established classes of DHODH (**Fig 10**). Sequences from several bacteria in the Pasteurellaceae family cluster within this group, suggesting for the first time that bacteria, such as *Haemophilus paracuniculus*, may have enzymes with DHUO activity. This also suggests that horizontal gene transfer may be the source of fungal DHUOs in a manner parallel to the appearance of class 1A DHODH in fungi [53].

Interestingly, DHUO activity seemed to vary significantly between species; enzymes from *C*. *bantiana* and *E*. *dermatitidis* supported the most robust growth of yeast while the *M*. *mycetomatis* counterpart did not reach comparable levels of growth. Further experiments are required to validate these differences in DHUO activity directly *in vitro*. Moreover, *the P*. *minimum* DHUO appeared more enzymatically active than that from *A*. *alternata*. Differences in DHUO activity may be caused by species requiring a different balance of pyrimidine metabolism and catabolism influenced by variation in activity of input enzymes. Previously, DHUOs were hypothesised to contribute to dihydropyrimidine detoxification [53] but it may be they also play a role in recycling of pyrimidines. Regardless of their role, these enzymes are not targeted by olorofim and as such, variations in dematiaceous mould susceptibility to the orotomide cannot be attributed to their DHUO.

While the phenomenon of having two different classes of DHODH does exist in select yeasts [42, 43], it appears that dematiaceous moulds are excluded from this. Further work is required to understand why certain species respond to olorofim and others appear more resistant as there are multiple possibilities for why this might be. Some species could have a stronger arsenal of efflux pumps that render them hardier in the face of xenobiotic compounds, as is seen for certain fungal species [93]. Perhaps local concentrations of olorofim never reach a threshold for target inhibition due to active export, rendering species less susceptible to the drug. Conversely, it may be differences in the target class 2 DHODH protein sequence that are responsible for reduced olorofim sensitivity. Indeed, this was shown to be the case for *Candida albicans* [4] and for instances of olorofim resistance where substitution mutation is the root cause [94]. Future work will address differences between class 2 DHODH proteins from susceptible and non-susceptible species. While the true cause for variation of olorofim sensitivity in dematiaceous moulds remains to be elucidated, it can be concluded that these species do not possess class 1A DHODH but do harbour DHUO in their repertoire of pyrimidine network enzymes.

## Supporting information

S1 Fig The yeast model is robust for exogenous class 1A DHODH. (A) Serial dilution assay of the negative control (β-gal^trunc^) and confirmed class 1A DHODH from *S*. *cerevisiae* (SC), *T*. *cruzi* (TC), *L*. *kluveryi* (LK1A) in *ura1Δ* cells in the presence of high copper concentration and pyrimidines. (B) Serial dilution assay of the negative control (β-gal^trunc^), confirmed class 1A DHODH from *S*. *cerevisiae* (SC) and confirmed class 2 DHODH from *A*. *fumigatus* (AF) in *ura1Δ* cells in the presence of high copper concentration and pyrimidines.
(TIF)

S2 Fig Purification of recombinant class 1A DHODH proteins. NuPAGE analysis of purified recombinant proteins. *T*. *cruzi* (TC), *L*. *kluveryi* (LK1A), *S*. *cerevisiae* (SC), *M*. *circinelloides* (MC) and *R*. *arrhizus* (RA).
(TIF)

S3 Fig Codon usage does not affect class 1A complementation. Serial dilution assay of the negative control (β-gal^trunc^), confirmed class 1A DHODH from *S*. *cerevisiae* (SC) and three putative *A*. *alternata* genes in *ura1Δ* cells. AA^Ec^, *E*. *coli* codon-optimised construct; AA^Nat^, native *A*. *alternata* sequence construct; AA^Sc^, *S*. *cerevisiae* codon-optimised construct.
(TIF)

S4 Fig Codon usage does not affect DHUO complementation. Serial dilution assay of the negative control (β-gal^trunc^), confirmed class 1A DHODH from *S*. *cerevisiae* (SC) and three putative *A*. *alternata* genes in *ura1Δ* cells. AA^Ec^, *E*. *coli* codon-optimised construct; AA^Nat^, native *A*. *alternata* sequence construct; AA^Sc^, *S*. *cerevisiae* codon-optimised construct in the presence of DHU.
(TIF)

S5 Fig Recombinant dematiaceous enzymes are not class 1A DHODH. (A) NuPAGE analysis of purified proteins. *A*. *alternata* (AA), *P*. *minimum* (PM). (B) Amount of DCIP reduced by proven and putative DHODH after 30 minutes. (C) Amount of FeCy reduced by proven and putative DHODH after 30 minutes. (D) Amount of orotate produced by proven and putative DHODH after 30 minutes.
(TIF)

S1 Table Entrez protein accession numbers used for analyses. (XLSX)

S2 Table List of yeast strains used in this study. (XLSX)

S3 Table BLASTP results for DHODH in Mucorales species. (XLSX)

S4 Table BLASTP results for DHODH in dematiaceous species. (XLSX)

S1 File Data for class 1A DHODH assays. (XLSX)

S2 File Data for inhibition of class 1A DHODHs. (XLSX)

S3 File Data for DHUO assays. (XLSX)

S4 File Data for inhibition of DHUOs. (XLSX)

S5 File Data for DHODH activity for all class 1A DHODHs and DHUOs. (XLSX)
