# Supplementary figures and images for: Characterisation of putative class 1A DHODH-like proteins from Mucorales and dematiaceous mould species
Source: PLoS One. 2023 Aug 2;18(8):e0289441. doi: 10.1371/journal.pone.0289441 (PMC10395836; doi:10.1371/journal.pone.0289441)

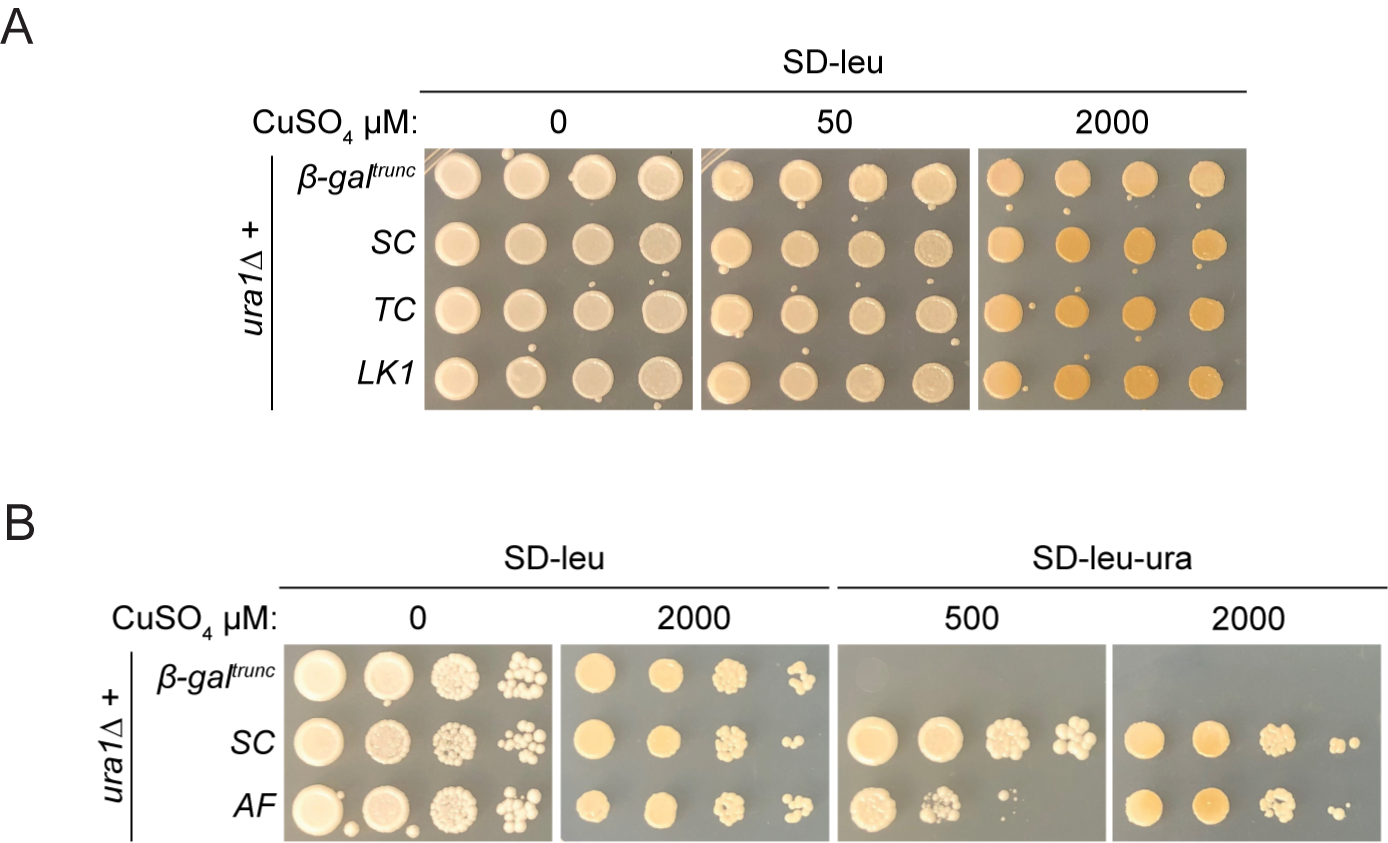

Supplement: S1 Fig — (A) Serial dilution assay of the negative control (β-galtrunc) and confirmed class 1A DHODH from S. cerevisiae (SC), T. cruzi (TC), L. kluveryi (LK1A) in ura1Δ cells in the presence of high copper concentration and pyrimidines. (B) Serial dilution assay of the negative control (β-galtrunc), confirmed class 1A DHODH from S. cerevisiae (SC) and confirmed class 2 DHODH from A. fumigatus (AF) in ura1Δ cells in the presence of high copper concentration and pyrimidines. (TIF) [file pone.0289441.s001.tif]

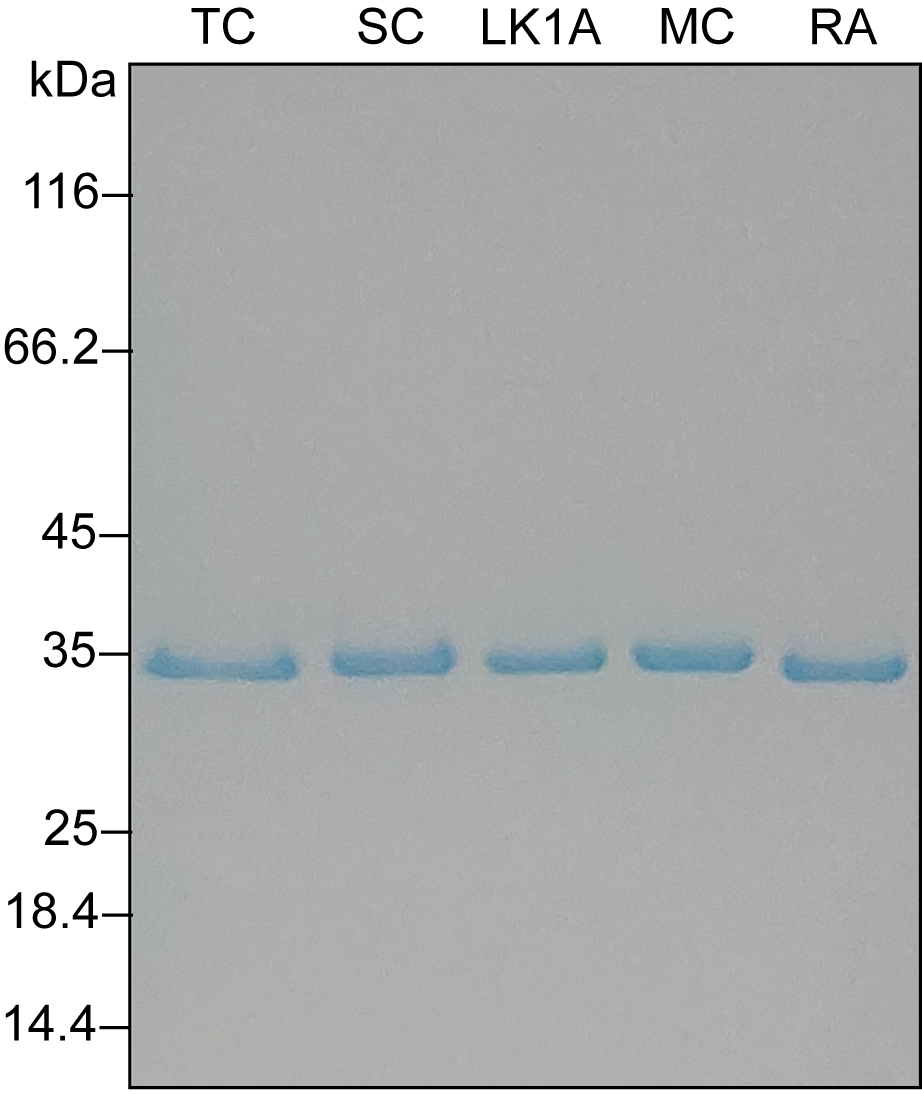

Supplement: S2 Fig — NuPAGE analysis of purified recombinant proteins. T. cruzi (TC), L. kluveryi (LK1A), S. cerevisiae (SC), M. circinelloides (MC) and R. arrhizus (RA). (TIF) [file pone.0289441.s002.tif]

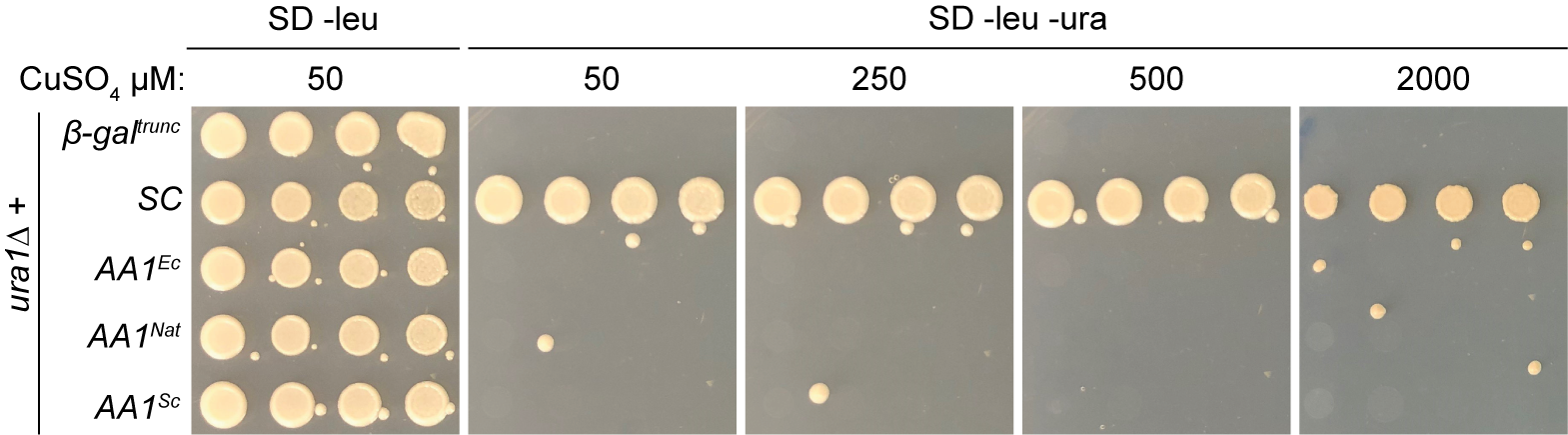

Supplement: S3 Fig — Serial dilution assay of the negative control (β-galtrunc), confirmed class 1A DHODH from S. cerevisiae (SC) and three putative A. alternata genes in ura1Δ cells. AAEc, E. coli codon-optimised construct; AANat, native A. alternata sequence construct; AASc, S. cerevisiae codon-optimised construct. (TIF) [file pone.0289441.s003.tif]

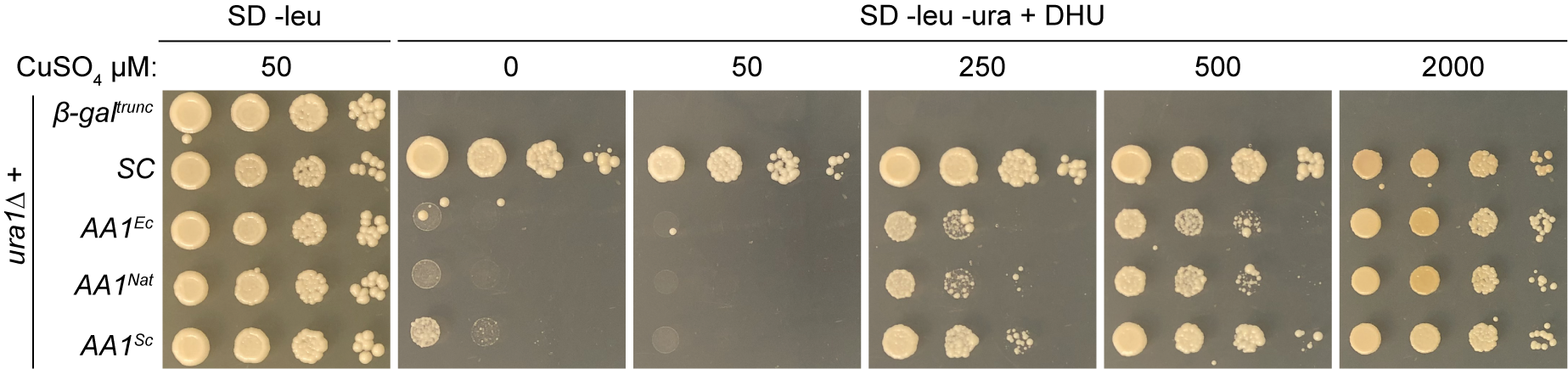

Supplement: S4 Fig — Serial dilution assay of the negative control (β-galtrunc), confirmed class 1A DHODH from S. cerevisiae (SC) and three putative A. alternata genes in ura1Δ cells. AAEc, E. coli codon-optimised construct; AANat, native A. alternata sequence construct; AASc, S. cerevisiae codon-optimised construct in the presence of DHU. (TIF) [file pone.0289441.s004.tif]

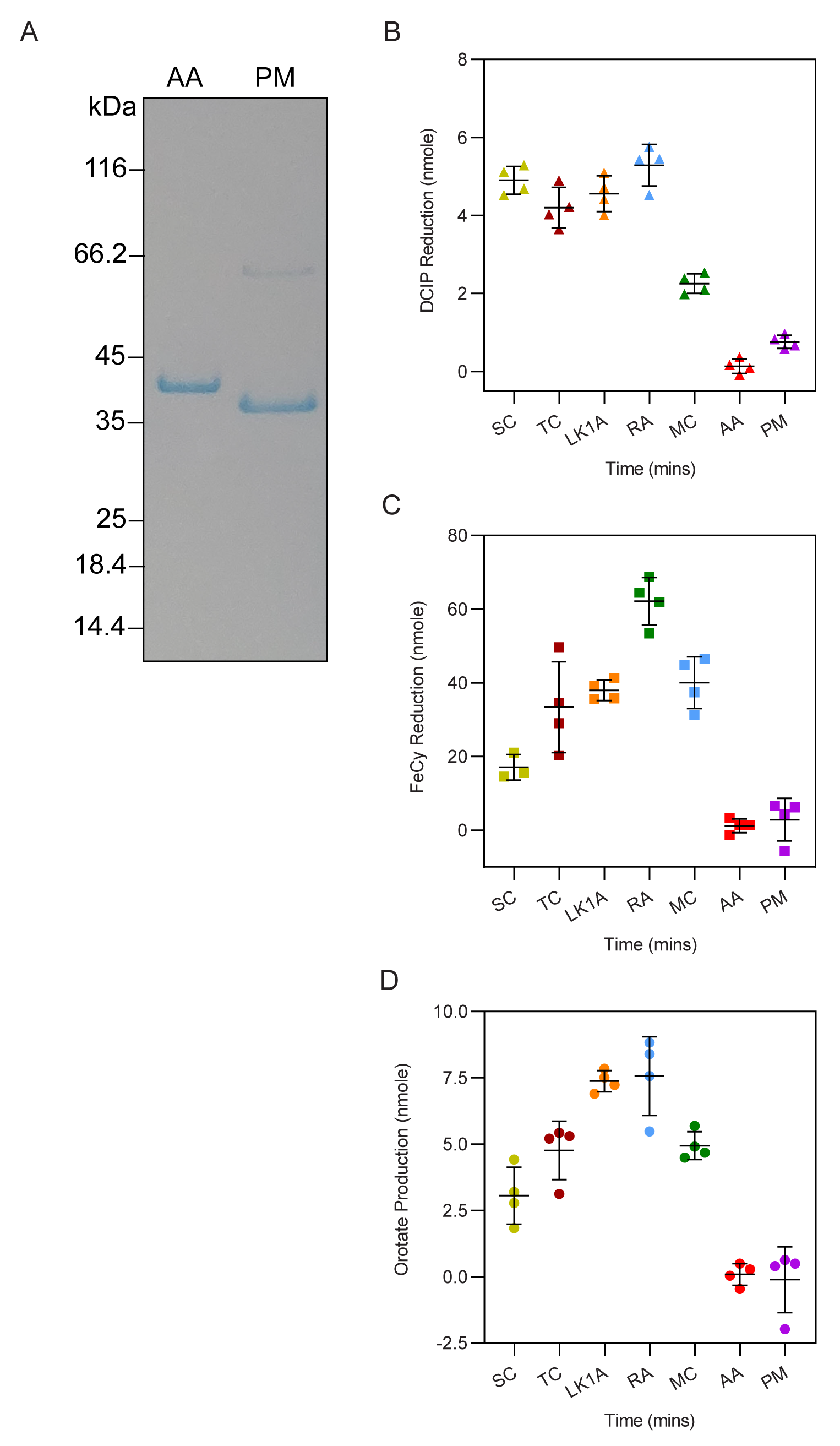

Supplement: S5 Fig — (A) NuPAGE analysis of purified proteins. A. alternata (AA), P. minimum (PM). (B) Amount of DCIP reduced by proven and putative DHODH after 30 minutes. (C) Amount of FeCy reduced by proven and putative DHODH after 30 minutes. (D) Amount of orotate produced by proven and putative DHODH after 30 minutes. (TIF) [file pone.0289441.s005.tif]
